# Epigenetic silencing of triple negative breast cancer hallmarks by Withaferin A

**DOI:** 10.18632/oncotarget.17107

**Published:** 2017-04-13

**Authors:** Katarzyna Szarc vel Szic, Ken Declerck, René A.J Crans, Jolien Diddens, David B. Scherf, Clarissa Gerhäuser, Wim Vanden Berghe

**Affiliations:** ^1^ Laboratory of Protein Chemistry, Proteomics and Epigenetic Signaling (PPES), Department of Biomedical Sciences, University of Antwerp, Antwerp, Belgium; ^2^ Workgroup Cancer Chemoprevention and Epigenomics, Division of Epigenomics and Cancer Risk Factors, German Cancer Research Center (DKFZ), Heidelberg, Germany; ^3^ Current address: Laboratory for GPCR Expression and Signal Transduction (L-GEST), Department of Biochemistry and Microbiology, University of Ghent, Ghent, Belgium; ^4^ Current address: Division of Hematology, Oncology and Stem Cell Transplantation, Center for Translational Cell Research, The University Medical Center Freiburg, Freiburg, Germany

**Keywords:** triple negative breast cancer, luminal breast cancer, epigenetics, Withaferin A, DNA methylation

## Abstract

Triple negative breast cancer (TNBC) is characterized by poor prognosis and a DNA hypomethylation profile. Withaferin A (WA) is a plant derived steroidal lactone which holds promise as a therapeutic agent for treatment of breast cancer (BC). We determined genome-wide DNA methylation changes in weakly-metastatic and aggressive, metastatic BC cell lines, following 72h treatment to a sub-cytotoxic concentration of WA. In contrast to the DNA demethylating agent 5-aza-2’-deoxycytidine (DAC), WA treatment of MDA-MB-231 cells rather tackles an epigenetic cancer network through gene-specific DNA hypermethylation of tumor promoting genes including ADAM metallopeptidase domain 8 (*ADAM8*), urokinase-type plasminogen activator (*PLAU*), tumor necrosis factor (ligand) superfamily, member 12 (*TNFSF12*), and genes related to detoxification (glutathione S-transferase mu 1, *GSTM1*), or mitochondrial metabolism (malic enzyme 3, *ME3*). Gene expression and pathway enrichment analysis further reveals epigenetic suppression of multiple cancer hallmarks associated with cell cycle regulation, cell death, cancer cell metabolism, cell motility and metastasis. Remarkably, DNA hypermethylation of corresponding CpG sites in *PLAU*, *ADAM8*, *TNSF12*, *GSTM1* and *ME3* genes correlates with receptor tyrosine-protein kinase erbB-2 amplification (HER2)/estrogen receptor (ESR)/progesterone receptor (PR) status in primary BC tumors. Moreover, upon comparing differentially methylated WA responsive target genes with DNA methylation changes in different clinical subtypes of breast cancer patients in the cancer genome atlas (TCGA), we found that WA silences HER2/PR/ESR-dependent gene expression programs to suppress aggressive TNBC characteristics in favor of luminal BC hallmarks, with an improved therapeutic sensitivity. In this respect, WA may represent a novel and attractive phyto-pharmaceutical for TNBC treatment.

## INTRODUCTION

Breast cancer (BC), which remains the leading cause of cancer related deaths in women, is a highly heterogeneous disease. High throughput microarray and sequencing-based gene expression profiling allowed identification of clinically applicable molecular BC subtypes with a different prognostic outcome, and translated into discovery of novel BC therapies. In a series of articles, Perou et al. and Sorlie et al. have identified five intrinsic molecular BC subtypes based on distinct gene expression patterns: “Normal breast-like”, estrogen receptor α positive (ESR+) “Luminal A” and “Luminal B”, and ER negative (ESR-) “Receptor tyrosine-protein kinase erbB-2 amplification positive (HER2+)/ESR-“ and “Basal-like” subtypes [1, 2]. The last one shares many features with BC referred to as “Triple negative breast cancer” (TNBC) (ESR-, progesterone receptor negative (PR-), HER2-), which constitutes 10% to 20% of all BCs. Compared to other BC subtypes, TNBCs grow more rapidly, are of higher grade and often have lymph node involvement at diagnosis. Moreover, women presenting with TNBC are younger, have poor prognosis and the shortest overall survival [2, 3]. To date therapeutic options for TNBC are limited to cytotoxic chemotherapy since no common molecular targets have been identified for this group so far [3, 4]. Besides changes in gene expression, extensive DNA methylation alterations also contribute to BC initiation and malignant transformation [5–8]. Several studies, including a comprehensive study by The Cancer Genome Atlas (TCGA) Network, have demonstrated that TNBCs are characterized by the most extensive hypomethylation, as opposed to luminal ESR+ cancers, which show the highest degree of hypermethylation [9–13]. Furthermore, differential DNA methylation patterns were shown to distinguish between TNBC patients with a better or worse prognosis [14]. Thus, DNA methylation not only aids the risk stratification of TNBC patients, but also due to its dynamic nature opens a new window of therapeutic opportunities in this highly aggressive form of BC.

The plasticity and potential reversibility of cancer-associated epigenetic changes in response to plant derived compounds has attracted much research interest with respect to development of novel cancer chemopreventive or therapeutic strategies [15, 16]. Throughout the years, various experimental models have demonstrated the ability of plant phytochemicals to change DNA methylation and histone acetylation marks, through changes in DNA methyltransferase (DNMT) and histone deacetylase (HDAC) activities (reviewed in [17–22]). As such, their molecular activities started to be compared to nucleoside analogues, such as 5-azacytidine (AZA) and 5-aza-2’-deoxyazacytidine (DAC), or histone deacetylase inhibitors, such as suberoylanilide hydroxamic acid (SAHA) and romidepsin, which are currently the only FDA-approved epigenetic drugs used in cancer treatment. However, it remains elusive whether phytochemicals evoke epigenetic changes by directly targeting chromatin and DNA methylation writer or eraser enzyme domains, or rather by indirectly interfering with metabolism and cell signaling pathways, which get finally transmitted to the epigenome [23, 24].

Withanolides extracted from *Withania somnifera (Ashwagandha* in Ayurvedic medicine), are known to be potent inhibitors of angiogenesis, inflammation, tumor development, and metastasis, and promoters of cardioprotection [25]. Many pharmacological studies have investigated the properties of *W. somnifera* in an attempt to authenticate its use as a multipurpose medical agent. The first described withanolide, Withaferin A (WA), has been extensively studied in BC *in vitro* as well as more recently *in vivo* models. Anticancer activity of WA was demonstrated at nanomolar concentrations in both ESR+ and ESR- BC models. Several important molecular targets were identified, such as vimentin (*VIM*), NFκB, β-tubulin, uPA (*PLAU*), interleukin-6 (*IL-6*), STAT3 or ESR, and more recently *in vivo* mouse models have confirmed WA efficacy in mammary tumors and BC xenografts [26–34]. Different placebo controlled human clinical trials with standardized *W. somnifera* extract with low doses of WA in healthy individuals did not reveal adverse toxicity and in general improved wellbeing [35, 36]. A phase II trial in BC patients with standardized *W. somnifera* extract with minor content of WA in combination with chemotherapy, reduced chemotherapy associated fatigue with a similar therapeutic outcome and without adverse toxic side effects [37]. Another phase II clinical trial is evaluating therapeutic efficacy of a formulation of *W. somnifera* extract containing WA in high grade relapsed or metastatic osteosarcoma patients (NCT00689195). However, controlled clinical studies in healthy individuals or cancer patients evaluating higher (therapeutic) doses of pure WA have not yet been reported. Also, whether prolonged WA treatment can elicit tumor suppression via epigenetic changes remains so far poorly characterized. Previously, we observed that WA exerts anticancer activity in part by changing chromatin accessibility at the *IL-6* promoter, a cytokine related to oncogenic, pro-inflammatory signaling in BC [38]. Moreover, we found that sub-cytotoxic WA concentrations which inhibit cancer metastasis reprogrammed transcription of several epigenetic enzymes regulating histone methylation in MDA-MB-231 and MCF-7 cells [34].

This prompted us to determine genome-wide epigenetic effects of WA in weakly-metastatic, epithelial-like MCF-7 and triple negative, aggressive MDA-MB-231 cells by Illumina 450k BeadChip arrays which quantify DNA methylation of more than 480 000 individual CpG dinucleotides scattered across the genome, and cover 99% of all RefSeq genes, including promoter related CpG islands (96%), CpG shores, and non-promoter methylation, in a cell population [39]. Verification of DNA methylation changes was performed by CpG bisulfite pyrosequencing and EpiTyper MassArray assays [40, 41]. Furthermore, complementary changes of histone marks for gene activation (H3K4me3) were investigated by chromatin immunoprecipitation (ChIP) analysis. Finally, WA induced DNA hypermethylation changes were compared with DNA methylation data from clinical breast cancer patient samples (TCGA database).

## RESULTS

### WA treatment does not elicit global DNA methylation changes in aggressive metastatic MDA-MB-231 human breast cancer cells

**Figure 1:**
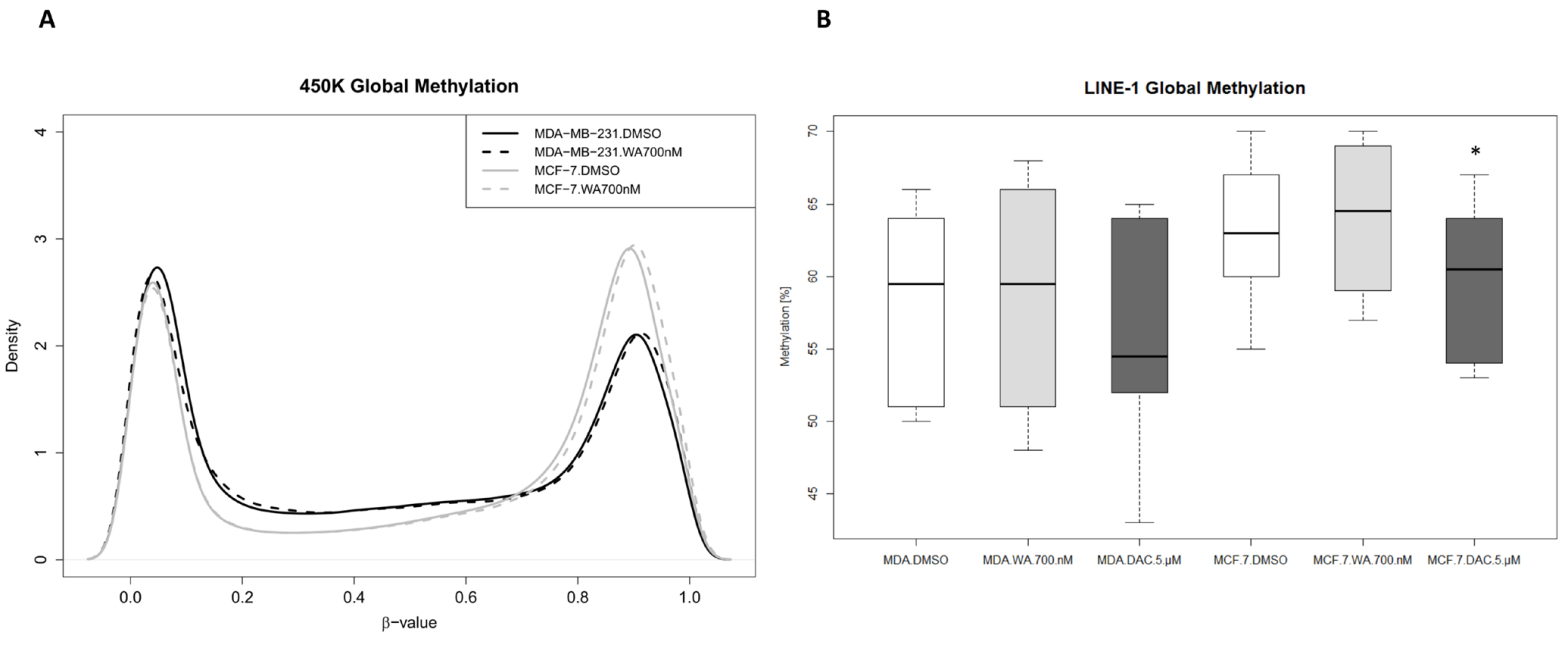
WA does not change global cell type specific DNA methylation (**A**) Global DNA methylation shifts were assessed by comparing the density plots based on β value distribution of all cg probes after quality filtering (second top box, Supplementary Figure 1) from the Infinium HumanMethylation450 BeadChip. MCF-7 cells (represented in gray) show a hypermethylated pattern as compared to MDA-MB-231 cells (shown in black). WA treatment does not result in global DNA methylation shifts (solid versus dashed lines) (**B**) LINE-1 methylation of MDA-MB-231 and MCF-7 cells treated with WA, DAC or DMSO control was determined by bisulfite pyrosequencing and represented as box and whisker plots. Whiskers represent maximum and minimum values, and the black bold line represents median methylation of at least two independent experiments. Statistically significant results of pair-wise comparisons (control/treatment) are marked with the asterisks (*p < 0.05) as calculated by Wilcoxon signed-rank test.

First we assessed whether strong suppression of metastasis and invasive properties of WA in triple negative MDA-MB-231 breast cancer cells observed upon 72 h exposure of MDA-MB-231 cells to sub-cytotoxic concentrations of WA (700 nM) can be explained by WA dependent epigenetic effects on DNA methylation [34]. Genome-wide changes in DNA methylation following WA treatment were quantified by Infinium Human Methylation450 BeadChip arrays in aggressive metastatic MDA-MB-231 cells and weakly metastatic MCF-7 cells. First, we visualized and compared global CpG loci density patterns from compound treated and solvent control cells using the density plots. WA treatment did not lead to major global methylation shifts in both of the studied cell lines. Remarkably, weakly metastatic MCF-7 cells were found to be clearly more methylated than highly metastatic MDA-MB-231 cells (Figure 1A). To confirm these global methylation degrees in independent sample sets before and after WA treatment as compared to the global DNA demethylating agent DAC, we next assessed the methylation status of long interspersed nucleotide elements (LINE-1), which serves as a surrogate marker of genome-wide methylation. Similarly to results obtained with 450k BeadChip analyses, methylation levels of LINE-1 elements were comparable before and after WA treatment, while DAC treatment decreased global methylation in both studied cell lines. Moreover, the higher methylation degree of MCF-7 cells as compared to MDA-MB-231 cells could also be reproduced by the LINE-1 assay (Figure 1B).

### WA induced gene silencing is associated with increased DNA methylation of their promoters in MDA-MB-231 cells

**Figure 2:**
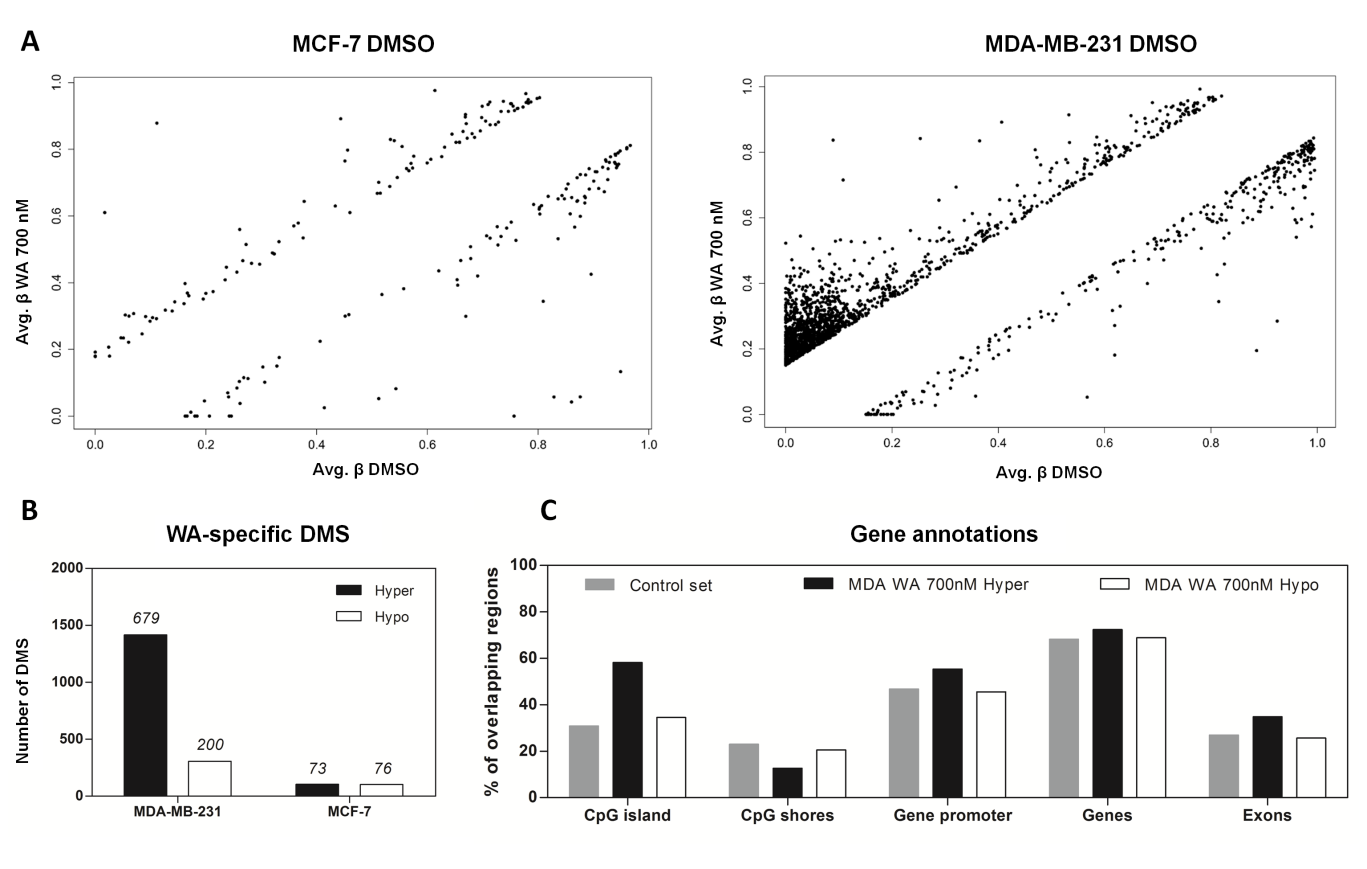
WA-specific DNA methylation alterations are more abundant in MDA-MB-231 cells in comparison to MCF-7 cells, with a preference for DNA hypermethylation of CpG islands and hypomethylation of CpG shores (**A**) Scatter plots represent differentially methylated sites (DMS) with a difference in CpG methylation of at least 15% between DMSO control and 700 nM WA treated MCF-7 and MDA-MB-231 cells. (**B**) The number of WA-specific CpG loci that showed increased (Hyper) or decreased (Hypo) CpG methylation of at least 15% relative to the solvent control samples. Above the bars the number of genes corresponding to the number of DMS is indicated. (**C**) The bar chart visualizes the percentage of CpG loci (y-axis) in the control data set (here represented as all cg probes on the 450k BeadChip) and WA-specific data sets, which overlap with different genomic compartments (CpG island, shore, gene promoter, gene body, exon) on the x-axis.

**Figure 3:**
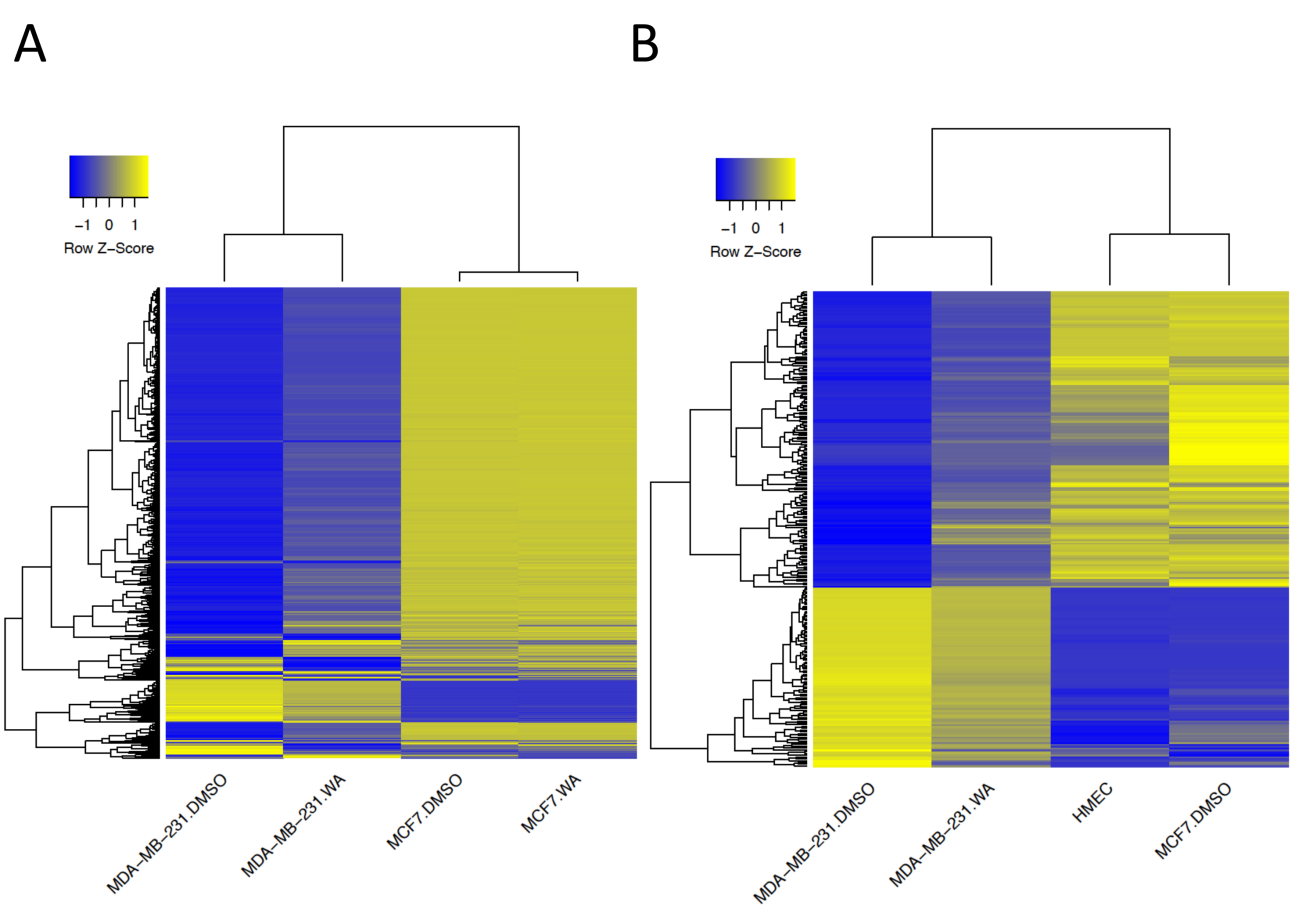
WA regulated CpG dinucleotides display distinct patterns of DNA methylation changes between MDA-MB-231 and MCF-7 cells (**A**) The heatmap representation of the methylation level (β-value) of 1,722 CpG sites with at least 15% methylation difference between control and WA treated MDA-MB-231 cells, compared to control and WA treated MCF-7 cells. (**B**) The heatmap representation of the methylation level (β-value) of 265 CpG sites which changed methylation in response to WA in MDA-MB-231 cells evolving towards the corresponding methylation status in MCF-7 and HMEC cells. β-values are represented as raw Z-scores, where the lowest methylation value is indicated in blue and the highest in yellow (0% - 100%).

Changes in DNA methylation following WA exposure involved both hyper- and hypomethylation (Figure 2A). To exclude possible false positive findings we considered CpG loci to be differentially methylated if a difference of ≥ 15% between WA treated and DMSO control was detected. In general, the number of differentially methylated sites (DMS) in MCF-7 cells was smaller (206 CpG sites corresponding to 149 genes) than in MDA-MB-231 cells (1722 CpG sites; 879 genes) (Figure 2B, Supplementary Figure 1), implying that epigenetic plasticity in MCF-7 cells is more restricted than in MDA-MB-231 cells. Interestingly, whereas a shift towards hypermethylation in MDA-MB-231 cells occurred mainly at previously unmethylated regions, hypomethylating effect was seen in highly methylated loci (Figure 2A). More careful investigation of hypermethylated CpG sites revealed that 887 DMS were found in CpG islands (CGI). Enrichment of WA hypermethylated CpG sites in CGI was also confirmed by the EpiExplorer web tool (+28,2% overlapping loci relative to the control set) (Figure 2C) [42]. Of special note, the majority of CpG sites whose methylation increased upon WA treatment in MDA-MB-231 cells were found to be hypermethylated in MCF-7 control cells. In contrast, most CpG sites whose methylation decreased upon WA treatment in MDA-MB-231 cells revealed hypomethylation in MCF-7 control cells (Figure 3A). Furthermore, comparing DNA methylation changes of WA-responsive genes in MDA-MB-231 cells with DNA methylation signatures of aggressive metastatic MDA-MB-231 cells versus weakly metastatic MCF-7 and normal breast epithelial HMEC cells (TCGA data set), we identified 265 hyper/hypomethylated CpG sites in response to WA, which promote a favorable methylation change, evolving towards the corresponding gene methylation status in weakly metastatic MCF-7 and normal HMEC cells (Figure 3B). Additional pathway enrichment analysis of the 265 CpG probe associated genes revealed WA silencing of ESR dependent cancer cell motility and proliferation [43–45]. In addition, network analysis further demonstrates silencing of the ErbB2 pathway involved in epithelial-to-mesenchymal transition (EMT) and glycolysis dependent breast cancer cell migration [46] (Supplementary Figure 2).

**Figure 4:**
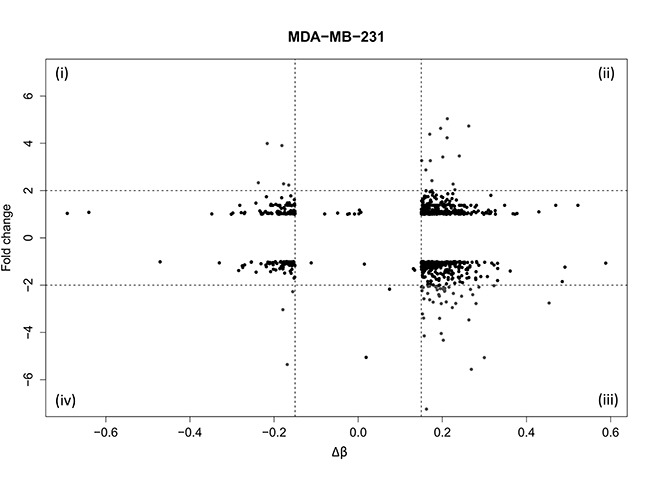
Identification of genes with coordinated DNA methylation and gene expression alterations in WA treated MDA-MB-231 cells Quadrant plot represents DNA methylation and gene expression of associated genes. The *x axis* represents mean methylation level (*β*-value) of all cg probes with ≥ 15% difference in methylation for the select target gene. On the *y axis* fold change of gene expression between control and WA treated MDA-MB-231 cells is indicated. Vertical dashed lines indicate the threshold of 15% methylation difference and horizontal dashed lines indicate a threshold corresponding to 2-fold gene expression change. The four quadrants represent genes (i) hypomethylated and upregulated, (ii) hypermethylated and upregulated, (iii) hypermethylated and downregulated and (iv) hypomethylated and downregulated in WA treated MDA-MB-231 cells.

To understand the biological relevance of DNA methylation changes induced by WA we next correlated the differential methylation patterns with previously published gene expression levels [34]. By applying stringent selection criteria for differential DNA methylation (Δβ ≥ 15%) and gene expression (FC ≥ ±2), we identified 67 genes in MDA-MB-231 and only 2 genes in MCF-7 cells (data not shown) for which DNA methylation inversely correlates with gene expression changes (Figure 4, quadrants (i), (iii)). These co-regulated genes in MDA-MB-231 cells were involved in cancer hallmark pathways related to cell cycle regulation, cell death, cancer cell metabolism, cell motility and metastasis (Supplementary Table 1). We observed silencing of various tumor promoting genes with WA induced DNA hypermethylation, including ADAM metallopeptidase domain 8 (*ADAM8*), urokinase-type plasminogen activator (*PLAU*), tumor necrosis factor (ligand) superfamily, member 12 (*TNFSF12*), as well as genes related to detoxification (glutathione S-transferase mu 1, *GSTM1*), or mitochondrial metabolism (malic enzyme 3, *ME3*). Reciprocally, we also identified few genes which become activated concomitantly with DNA hypomethylation following WA treatment, such as genes involved in L-cysteine and redox metabolism i.e. glutaredoxin 2 (*GLRX2*) and glutamine-fructose-6-phosphate transaminase 2 (*GFPT2*), syntaxin11 (*STX11*) involved in vesicular trafficking, and inducible nerve growth factor (*VGF*) involved in suppression of cancer progression and metastasis [47, 48]. Finally, upon integrated pathway analysis of the co-regulated genes (Supplementary Tables 1, 2), 4 major networks could be identified related to cell viability, cell motility, cell proliferation and cell-cell signaling, which are interconnected through NFκB, ESR1 as well as MAPK/PI3K/Akt kinase families (Supplementary Figures 3–6, Supplementary Table 2).

### Verification of WA induced DNA hypermethylation and gene expression alterations reveals epigenetic reprogramming of *ADAM8*, *PLAU*, *TNFSF12*, *ME3* and *GSTM1* target genes, associated with HER2/PR/ESR status in TNBC

**Figure 5:**
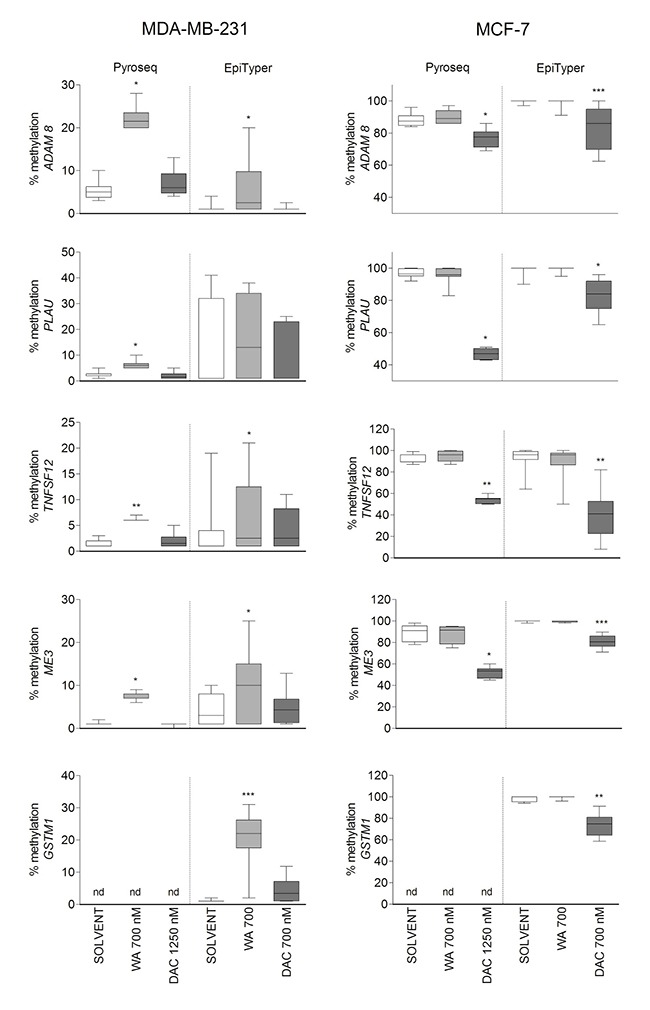
Verification of WA induced DNA hypermethylation The box plots in the left panel represent median pyrosequencing (Pyroseq) and EpiTyper MassArray (EpiTyper) DNA methylation levels of all representative CpG dinucleotides/units present in the sequenced regions of interest in MDA-MB-231 cells. In the right panel box plots of median Pyroseq and EpiTyper methylation levels in MCF-7 cells are shown for *ADAM8*, *PLAU*, *TNFSF12*, *ME3* and *GSTM1* genes. Data represent results from two (Pyroseq) or three (EpiTyper) technical replicate experiments. For both panels statistically significant results of pairwise comparisons (control/treatment) for each cell line are indicated with the asterisks (*p < 0.05; **p < 0.01; ***p < 0.001) as calculated by Wilcoxon signed rank test.

**Figure 6:**
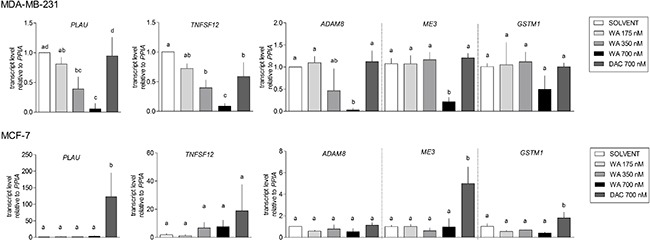
Verification of gene expression alterations in WA treated MDA-MB-231 and MCF-7 cells Relative gene expression levels were determined by a real-time quantitative PCR and normalized to the cyclophilin housekeeping gene (*PPIA*). 2^-ΔΔCt^ method was used for calculations. Bar graphs represent relative mRNA (mean ± SEM) levels of three independent experiments, each performed in triplicate. The bars marked with different letters are significantly different (*p* < 0.05) as determined by one-way ANOVA (Tukey Multiple Comparison posttest).

Because WA predominantly increased DNA methylation, we mainly focused on verification of hypermethylation effects obtained for selected genes related to cancer invasiveness (*ADAM8*, *PLAU*, and *TNFSF12*), detoxification (*GSTM1*) or mitochondrial functions (*ME3*), in relation to gene expression silencing. WA induced hypermethylation changes were verified by pyrosequencing and EpiTyper MassArray approaches on matched independent bisulfite-modified samples. For a gene-specific assay design we first looked at the methylation distribution in the whole gene locus of the selected BC candidate genes in both cell lines, based on available 450k probes (Supplementary Figure 7). We next designed the pyrosequencing and/or EpiTyper assays in a gene region with the biggest DNA methylation difference between MDA-MB-231 and MCF-7 cells, which was also strongly regulated by WA (cg probes in red frames in Supplementary Figure 7). We could successfully reproduce large methylation differences between weakly metastatic ESR+ MCF-7 and aggressive metastatic ESR- MDA-MB-231 cells. Also, methylation differences between WA treated and control samples observed in 450K analyses, could be verified by EpiTyper MassArray and pyrosequencing assays, although the effect size was more pronounced in 450k analyses (Figure 5). At the transcriptional level, hyper- or hypomethylation of *ADAM8*, *PLAU*, *TNFSF12, ME3* and *GSTM1* genes inversely correlated respectively with low or high expression levels (Figure 6). Finally, as shown for all five genes, treatment with 700 nM DAC led to a strong demethylating effect in MCF-7 cells, which was associated with increased, however not significant in all instances, expression of these genes (Figures 5 and 6).

**Figure 7:**
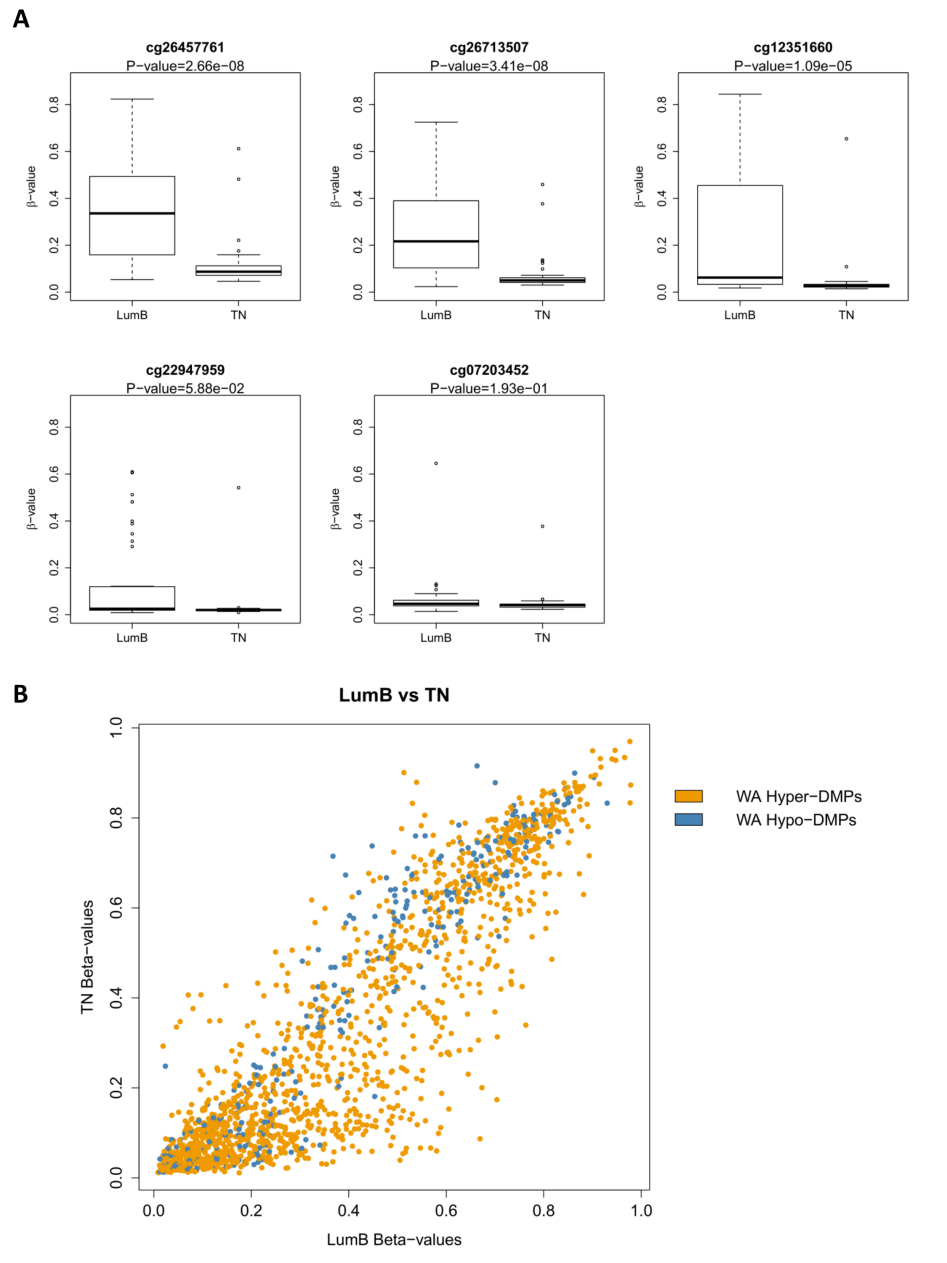
DNA methylation of verified PLAU CpG sites correlates with triple negative (TN) and Luminal B PAM50 intrinsic subtypes in primary breast tumor samples (**A**) The box plots represent median methylation level of the selected CpG sites in the *PLAU* gene in Luminal B and TN primary breast tumor samples as determined by Illumina 450k BeadChip arrays based on the dataset from [11]. Sample identifiers are provided in Supplementary Table 3. Benjamini-Hochberg-adjusted *P*-values are indicated above each box plot. *P*-value < 0.05 was considered significant as determined by Wilcoxon signed-rank test. (**B**) Scatter plot comparing methylation levels in luminal B and TN primary breast tumor samples of the WA regulated DMS. Hyper-DMS induced by WA are indicated in orange and Hypo-DMS are indicated in blue.

We next asked whether any of the verified CpG sites of *ADAM8, PLAU, TNFSF12, ME3* and GSTM1 were differentially methylated in invasive BC patient samples from TCGA database (sample identifiers are provided in Supplementary Table 3). *PLAU* DNA hyper- or hypomethylation at selected CpG sites (underlined in Table 1) correlated respectively with luminal B and TNBC intrinsic subtypes (Figure 7A). Along the same line, upon comparing WA responsive hyper- or hypomethylated CpG sites of MDA-MB-231 cells with corresponding DNA methylation levels observed in TNBC and luminal B BC patient samples, we observed a predominant overlap with the DNA hypermethylation signature of luminal BC as compared to TNBC patients (Figure 7B). Subsequent analysis of invasive BC patient samples (*n* = 871) by means of MEXPRESS software revealed significant correlations between DNA methylation levels of the verified CpG motifs and the HER2, ESR and PR status of BC patients (Table 1) [49]. In this respect, WA induced DNA hypermethylation of multiple CpG sites may contribute to epigenetic reprogramming of genes involved in aggressiveness of TNBC towards less aggressive luminal-like BC hallmarks.

**Table 1:** Correlation of *ADAM8*, *PLAU*, *TNFSF12*, *ME3* and *GSTM1* DNA methylation with HER2/PR/ESR status in breast carcinoma patients

Gene	cg probe	HER2 status	PR status	ER status
***ADAM8***	cg24382527	4,18E-01	4.9E-01	6.04E-03
	cg15460093	8,22E-01	6.2E-01	7.61E-04
***PLAU***	cg26457761	4,12E-05	2.8E-11	2.20E-16
	cg26713507	5,43E-03	3.1E-11	2.20E-16
	cg12351660	1,24E-01	2.5E-02	3.32E-04
	cg22947959	1,44E-01	2.1E-03	3.32E-04
	cg07203452	8,02E-01	6.3E-03	4.22E-02
***GSTM1***	cg24506221	9,98E-01	4.0E-01	4.29E-01
	cg18938907	5,13E-01	1.3E-02	9.37E-05
	cg17901463	2,92E-01	3.9E-04	1.70E-06
***TNFSF12***	cg09801082	6,62E-01	2.2E-16	2.20E-16
	cg06479877	3,56E-02	4.3E-03	1.99E-04
	cg26706811	5,48E-01	2.5E-08	4.51E-09
***ME3***	cg14881618	4,18E-03	1.2E-03	3.24E-04
	cg08387285	6,00E-02	1.2E-09	2.92E-12
	cg11953383	6,00E-02	3.9E-09	2.33E-10
	cg20334849	7,26E-02	2.4E-10	5.78E-13
				Bold italic values *p* value < 0.05 (< 5.00 E-02)

Gene specific CpG DNA methylation levels (ADAM8. PLAU. TNFSF12. ME3 and GSTM1) in clinical data of primary breast tumors (*n* = 871. The Cancer Genome Atlas) were correlated to HER2. ESR or PR status by MEXPRESS [11. 49]. Benjamini-Hochberg-adjusted *p* value < 0.05 was considered significant as determined by a non-parametric Wilcoxon signed-rank test.

### WA induced DNA hypermethylation coincides with chromatin silencing at the *PLAU* gene promoter

**Figure 8:**
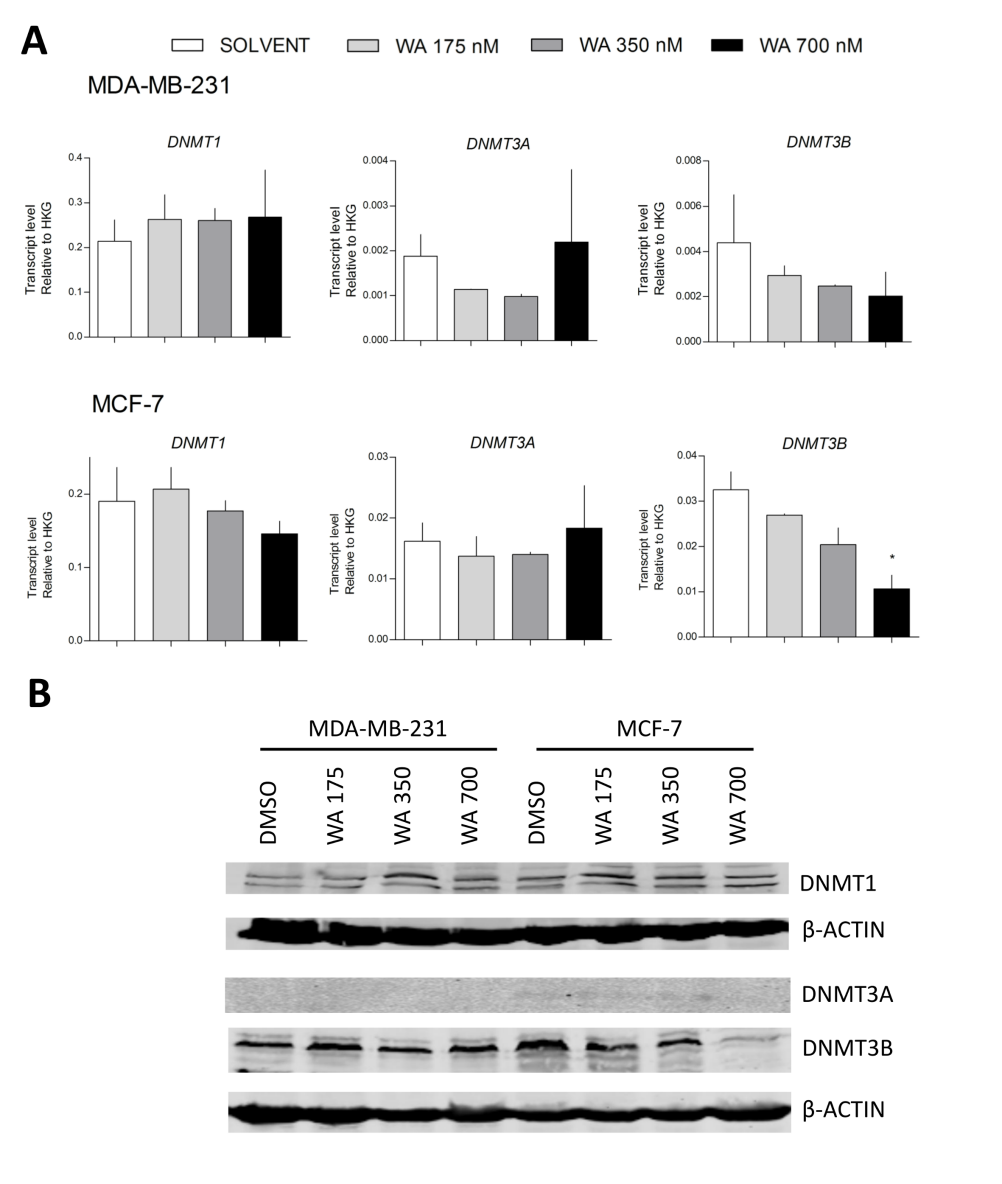
WA does not alter DNMTs gene and protein expression (**A**) Effect of WA on *DNMT1*, *DNMT3A* and *DNMT3B* gene expression in MDA-MB-231 and MCF-7 cells normalized to select housekeeping genes and relative to DMSO-treated MCF-7 sample (2^-ΔΔCt^) as determined by Human Epigenetic Chromatin Modification Enzyme qPCR Array as previously described. Bars represent *DNMT1*, *DNMT3A* and *DNMT3B* relative mRNA (mean ± SEM) expression of at least two independent experiments. (**B**) Effect of WA on *DNMT1*, *DNMT3A* and *DNMT3B* protein expression in MDA-MB-231 and MCF-7 cells, as compared to to β-Actin protein levels.

**Figure 9:**
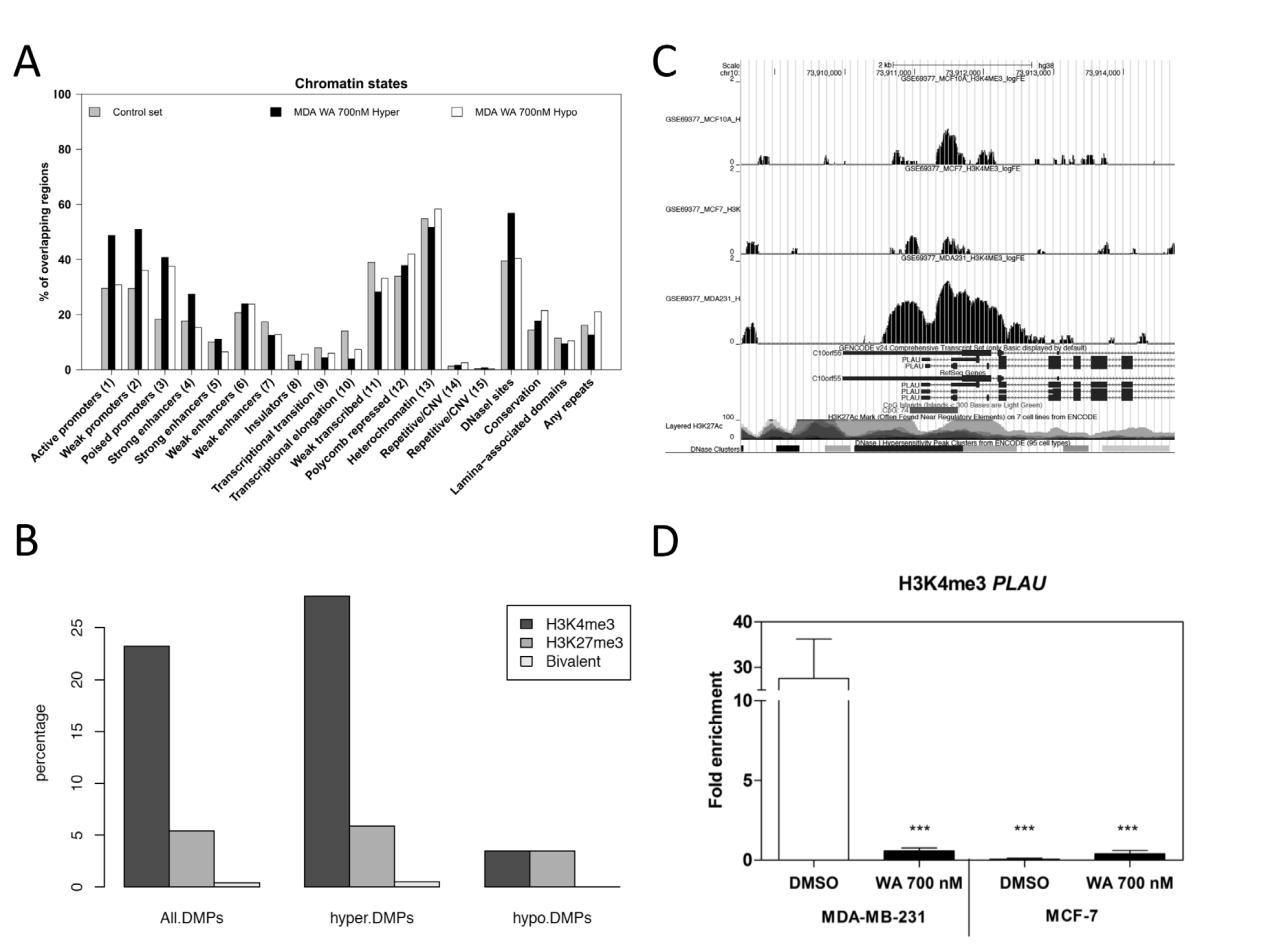
WA treatment decreases gene specific H3K4me3 activation marks (**A**) The bar chart visualizes the percentage of CpG loci (y-axis) in the control data set (here represented as all cg probes on the 450k BeadChip) and WA-specific data sets, which are enriched in specific chromatin states (x-axis). (**B**) Bar charts representing the percentage overlap between WA-induced differentially methylated positions (DMPs) and H3K4me3, H3K27me3 histone marks in MDA-MB-231 cells [54, 55]. Overlap was performed for all the 1722 WA-induced DMPs, including hypermethylated DMPs (hyper-DMPs) and hypomethylated DMPs (hypo-DMPs).(**C**) UCSC genome browser view of the PLAU promoter visualizing H3K4me3 ChIP data in normal MCF-10A, low metastatic MCF-7 and aggressive metastatic MDA-MB-231 cells. [54, 55]. (**D**) Effect of WA on H3K4me3 active chromatin mark in MDA-MB-231 and MCF-7 cells was evaluated by chromatin immunoprecipitation (ChIP), using Chromatrap Pro A kit. The enriched DNA was quantified by real-time PCR using promoter-specific primers. Bar plots represent fold enrichment of H3K4me3 mark relative to the FLAG mock antibody control. Results represent mean ± SD of at least two independent experiments. Statistical significance between MDA-MB-231 DMSO control versus WA treated MDA-MB-231, DMSO and WA treated MCF-7 cells is indicated with asterisks (***p < 0.0001) and was determined by ANOVA (Tukey Multiple Comparison posttest).

Despite gene specific hypermethylation differences following WA treatment, we were not able to demonstrate a significant increase in gene transcription and/or protein expression of any of the DNMTs in MDA-MB-231 cells (Figure 8A–8B). However, we cannot exclude indirect regulation of DNMT activity through post-translational modifications, microRNAs and/or additional chromatin (HDAC, SIRT) complexes [50–53]. Taking into account rather moderate WA-responsive DNA methylation changes in comparison to the relative strong gene silencing effects observed for *PLAU* and other tumor promoting genes, we next explored whether WA treatment changed chromatin marks which can synergize with DNA hypermethylation to mediate strong gene silencing. In line with the WA-specific decrease of chromatin accessibility observed at a DNAse hypersensitive site at the *IL6* gene locus [38], the EpiExplorer web tool further revealed significant enrichment of WA induced hypermethylation at DNase hypersensitive sites, promoters and enhancers (Figure 9A) [42]. In addition, upon comparing WA hypermethylated genes to the oncofetal bivalent breast cancer signature of H3K4me3 and H3K27me3 marks, we observed a predominant overlap with H3K4me3 marks (Figure 9B) [54, 55]. Of particular interest, ChIP-seq experiments revealed strong genome-wide gain of H3K4ac and H3K4me3 at gene promoters associated with breast cancer-related phenotypic traits, such as estrogen response and epithelial-to-mesenchymal transition pathways [54, 55]. Upon zooming in at the *PLAU* specific CpG region which gets hypermethylated following WA treatment, we observe strongly increased levels of H3K4me3 ChIP signal in aggressive metastatic MDA-MB-231 cells in comparison to low metastatic MCF-7 and normal MCF-10A cells (Figure 9C) [54, 55]. Along the same line, we performed ChIP to investigate changes in H3K4me3 gene activation marks across the promoter region of *PLAU* in MDA-MB-231 and MCF-7 cells, to verify whether WA specific reduction of uPA expression as well as decreased invasiveness of MDA-MB-231 cells corresponds with decrease in H3K4me3 levels [34]. In highly metastatic MDA-MB-231 cells which strongly express *PLAU*, H3K4me3 ChIP analysis confirmed an open chromatin structure, strongly enriched for the H3K4me3 gene activation mark at the hypomethylated *PLAU* promoter region. In contrast, the hypermethylated *PLAU* gene promoter region was depleted of H3K4me3 in weakly metastatic MCF-7 cells, in which *PLAU* gene was not expressed. Moreover, a significant decrease in this active mark was observed in MDA-MB-231 cells treated with WA, which may mark the promoter for DNA hypermethylation concomitantly with strong transcriptional silencing (Figure 9D).

**Figure 10:**
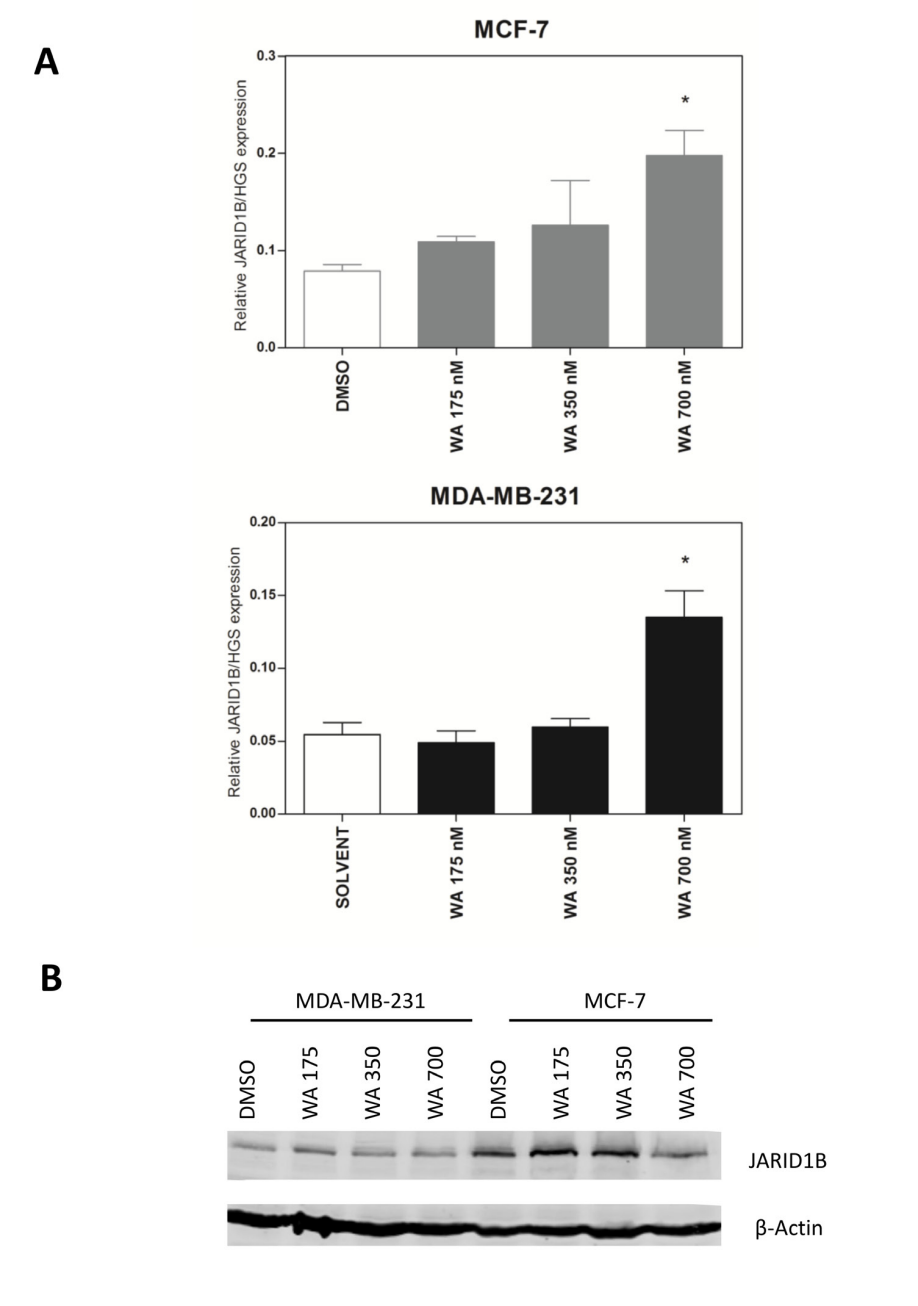
WA treatment increases JARID1B (KDM5B) mRNA, but not protein expression (**A**) Effect of WA treatment on *KDM5B* gene expression in MCF-7 and MDA-MB-231 cells normalized to selected housekeeping genes as determined by the Human Epigenetic Chromatin Modification Enzyme qPCR Array as previously described [34]. Statistical significance between DMSO controls versus WA treatments for each gene is indicated with asterisks (*p < 0.05). (**B**) Effect of WA treatment on JARID1B protein expression in MCF-7 and MDA-MB-231 cells, as compared to β-actin protein levels.

Since previous findings identified the jumonji-domain histone demethylases JARID1B (*KDM5B*) responsible for specific removal of H3K4me3 active chromatin among the most WA regulated epigenetic erasers [34], we further checked by qPCR and Western Blot analysis whether WA specific changes in *KDM5B* transcription or protein expression levels could explain the observed reduction in H3K4me3 levels in MDA-MB-231 cells upon WA treatment. However, whereas cell type specific differences in H3K4me3 levels (high in MDA-MB-231, low in MCF-7) correspond with opposite JARID1B expression levels (low in MDA-MB-231, high in MCF-7), no significant WA treatment specific changes in JARID1B protein expression could be observed to explain gene specific reduction of H3K4me3 levels at the *PLAU* promoter (Figure 10A–10B). Based on these results, we assume that WA-dependent gene silencing of *PLAU* in MDA-MB-231 cells may require concerted action of several chromatin complexes (KDM-family members, LSD1, NuRD, PARP1), post-translational modifications (SUMO) and/or noncoding RNAs (miR-137, miR-448, lcRNA MALAT1) to suppress metastasis programs in response to WA [55–65]. Alternatively, we cannot exclude that WA induced oxidative stress pathways may promote catalytic activity of JARID1B enzymes to decrease H3K4me3 levels [63, 66].

## DISCUSSION

WA holds promise for further preclinical development in BC treatment because it exerts strong anticancer activity, both *in vitro* as well as *in vivo*, at pharmacologically achievable concentrations [26, 31, 33, 34, 67]. Recent evidence indicates that natural chemopreventive and therapeutic agents can potentially reverse adverse epigenetic marks in cancerous cells by influencing the activity or expression of DNMTs and histone modifying enzymes (e.g. HDACs or sirtuins), leading to decreased cancer cell proliferation, while exhibiting no or limited cellular toxicity. In the present study we have investigated whether anticancer effects following prolonged treatment to sub-cytotoxic concentrations of WA involve epigenetic regulation in MCF-7 and MDA-MB-231 cells. We studied global as well as gene-specific changes in DNA methylation and chromatin remodeling events following WA exposure of BC cells with varying metastatic properties.

By two independent methods, namely Illumina 450k BeadChip arrays and LINE-1 pyrosequencing, we demonstrated that WA did not result in widespread methylation alterations in both studied cell lines, in contrast to effects observed by a global demethylating agent DAC (Figure 1). Besides WA specific epigenetic effects, we also demonstrate that triple negative MDA-MB-231 cells show a clearly lower level of global methylation as compared to MCF-7 cells, in line with the observations in triple negative, and luminal breast tumors. However, even if cell line models largely retain the features of primary tumors we also acknowledge existence of epigenetic discrepancies between them and highlight the importance of verifying the clinical relevance of these findings in breast cancer patient cohort studies [68].

Stringent selection criteria were applied to our 450k BeadChip datasets to exclude possible false positive findings and to identify target genes with differential DNA methylation for further verification by pyrosequencing and Epityper MassArray. More particularly, CpG loci were selected whose DNA methylation varied ≥ 15% between WA and control sample, and for which at least two proximal CpG sites from the same gene were differentially methylated (Supplementary Figure 1). By doing so we demonstrated that gene-specific WA effects on DNA methylation were bidirectional, involving both hyper- and hypomethylation of important BC related genes. The majority of genes showed increased level of cytosine methylation within CGI, especially in MDA-MB-231 cells (Figure 2). Interestingly, WA specific DNA methylation targets in MDA-MB-231 cells showed a clearly opposite pattern of DNA methylation in MCF-7 cells or normal HMEC cells. While they were strongly hypomethylated in aggressive metastatic MDA-MB-231 cells and showed increased DNA methylation following WA treatment, they were almost fully methylated in weakly metastatic MCF-7 cells (Figure 3A), as well as normal breast epithelial HMEC cells (Figure 3B). The reverse pattern was observed for WA hypomethylated genes (Figure 3). This was confirmed by pyrosequencing and EpiTyper MassArray for several targets, in particular *PLAU*, *ADAM8*, *TNFSF12*, *ME3* and *GSTM1* genes (Figure 5). More importantly, mRNA expression of these genes inversely correlated with methylation status, which suggests an important role for DNA methylation in their regulation (Figures 4, 5, and 6). Further comparative pathway analysis of cell type (HMEC, MCF-7, MDA-MB-231) and/or WA specific DNA methylation differences revealed that WA simultaneously elicits DNA methylation and gene expression changes in MDA-MB-231 cells associated with multiple cancer hallmarks (Supplementary Tables 1, 2), and cancer related networks related to cell survival, motility, proliferation and cell-cell signaling (Supplementary Figures 2–6).

Remarkably, upon comparison of clinical DNA methylation data from the TCGA with WA induced DNA hypermethylation of CpG motifs in *PLAU*, *ADAM8*, *TNFSF12*, *ME3* and *GSTM1* genes, we identified a strong correlation with the HER2/PR/ESR status in invasive BC patients (Table 1, Figure 7A). Moreover, overlapping WA-responsive hypermethyated CpGs with DNA methylation signatures of HER2/PR/ESR positive luminal BC and HER2/PR/ESR negative TNBC patients revealed more pronounced clustering of WA hypermethylated CpGs to epigenetic methylated targets in luminal BC with an improved therapeutic sensitivity (Figure 7B). At the pathway level, our results suggest intimate crosstalk between regulation of DNA methylation, hormone receptor, and NFκB specific (tyrosine) kinase signaling pathways by WA in BC, in line with previous reports [38, 69–71] (Supplementary Figures 2–6).

Although numerous studies have focused at DNA demethylating effects of plant compounds, analogous to DAC or AZA effects, we show here that hypermethylating effects of WA contribute to the overall anticancer activity of this compound, without increasing DNMT mRNA or protein levels (Figure 8A–8B). In contrast, Mirza et al. [72] observed decreased DNMT expression at WA doses of 8 and 10 μM for MCF-7 and MDA-MB-231, respectively, which exceed the IC_50_ toxicity values for 72h WA exposure [32–34, 73]. It is thus likely that in the latter case, DNMT downregulation might represent a secondary effect of WA induced cytotoxicity and cell death. Alternatively, WA-specific metabolic changes could also trigger dose dependent effects in chromatin remodeling and DNA hypermethylation [26, 74, 75]. Of particular interest, we observed WA-specific epigenetic regulation of the folate metabolizing enzyme cystathionine-β-synthase (*CBS*), which may indirectly modulate DNA methylation dynamics through perturbation of the metabolic S-adenosylmethionine (SAM) flux in the one-carbon metabolism pathway, and which deserves further investigation [76, 77].

Furthermore, in agreement with previous observations which demonstrate a WA-specific decrease of chromatin accessibility at the *IL6* gene locus [38], we show that WA induced DNA hypermethylation is associated with decreased active H3K4me3 chromatin marks at the *PLAU* gene promoter to levels observed in weakly metastatic MCF-7 or MCF-10A cells (Figure 9C–9D). Along the same line, H3K4me3 was found to be mutually exclusive with *de novo* DNMT activity [78]. However, at this stage it is difficult to infer whether WA induced gene silencing is primarily caused by JARID1B specific histone H3K4me3 demethylation, DNA hypermethylation, their combined effect, or requires additional DNA/chromatin remodeling complexes (e.g. LSD1/NuRD, KDM5 isoforms or other KDM demethylases) and/or non-coding RNAs (miR-137, miR-448, lcRNA MALAT1) since WA specific changes in JARID1B transcription were not confirmed at the protein expression level (Figure 10B) [55–62]. The function of JARID1B is complex, and conflicting results have been published. It has been described as a tumor suppressor gene to inhibit breast cancer metastasis and angiogenesis through silencing of chemokine expression, in line with our results [79]. Furthermore, upregulation of JARID1B was also found to promote genome stability or tumor senescence [80–82]. Reciprocally, besides tumor suppressor functions of JARID1B, oncogenic effects in luminal breast cancer have recently also been described [83] by SUMO/ubiquitin modified JARID1B forms [58, 84]. Since WA was also demonstrated to induce senescence in induced cancer stem cell-like iCSCL-10A model it is plausible that JARID1B may mediate this effect [85]. However, additional epigenetic editing experiments might be required to fully untangle gene context dependent crosstalk of JARID1B with DNA methylation to fully understand WA-specific epigenetic reprogramming of HER2/PR/ESR dependent gene expression programs in TNBC [86, 87]. More particularly, by coupling epigenetic effector domains to *PLAU* specific CRISPR-Cas9 guide RNAs it might become feasible to therapeutically silence *PLAU* dependent breast cancer cell motility-metastasis properties in TNBC [86–89].

In conclusion, our results demonstrate that WA-induced DNA hypermethylation and H3K4me3 demethylation suppress *PLAU* gene expression and aggressive TNBC characteristics in favor of luminal BC hallmarks with an improved therapeutic sensitivity. In this respect, WA may represent a novel and attractive phyto-pharmacological compound to overcome therapy resistance in TNBC.

## MATERIALS AND METHODS

### Reagents

Working solution of Withaferin A (purity ≥ 97%, purchased from Altavista Phytochemicals Pvt Ltd.) was prepared as previously described [34]. 22 mM DAC (Sigma Aldrich, St. Louis, MO, USA) stock was prepared in DMSO and further diluted in complete growth medium to a final concentration immediately before use. Tri-Methyl-Histone H3 (Lys4) (C42D8) Rabbit monoclonal antibody (Cat.no. #9751, ChIP grade) was purchased from Cell Signaling Technology (Danvers, MA, USA). GelRed™ Nucleic Acid Stain in water (10 000 x Stock) was purchased from Biotium (Hayward, CA, USA).

### Cell lines and cell culture

The MDA-MB-231 and MCF-7 cell lines, purchased from American Type Culture Collection (Manassas, VA, USA), were cultivated and treated with WA and DMSO as previously described [34]. DAC freshly diluted in medium was added every 24 hours for a total time of 72 hours. Both cell lines have successfully been authenticated by short tandem repeat (STR) profiling (Cell ID System, Promega, Madison, WI, USA) according to the manufacturer's instructions. Prior to experiments with WA and DAC cell growth properties were evaluated by means of *xCELLigence* system (Roche, Penzberg, Germany) as previously described [34]. Mycoplasma contamination status was routinely monitored by the use of MycoAlert Mycoplasma Detection Kit (Lonza Group, Basel, Switzerland). All cell culture reagents were purchased from Life Technologies (Praisley, UK).

### DNA extraction and bisulfite conversion

Genomic DNA (gDNA) from MDA-MB-231 and MCF-7 cells, treated with the compounds as previously described [34], was isolated with DNeasy Blood & Tissue kit (Qiagen Hilden, Germany). 1000 ng of gDNA was bisulfite converted using the EZ DNA methylation kit (Zymo Research, Orange, CA, USA) according to manufacturer's instructions. Successful bisulfite conversion was confirmed by the amplification of a 208 bp amplicon of the *SALL3* gene (95 °C 15 min; then 45 cycles of 94 °C 30 sec, 55 °C for 30 sec, 72 °C for 30 sec; followed by 72 °C for 10 min) using the primer set provided in Supplementary Table 4. Additional internal sequence-specific bisulfite conversion controls were included in the pyrosequencing target gene assays.

### Infinium human methylation450K beadchip array

Genome-wide DNA methylation was analyzed on Infinium HumanMethylation450 BeadChip platform (Illumina, San Diego, CA, USA) at the DKFZ Genomics and Proteomics Core Facility. 4 μl of bisulfite-converted DNA (~150 ng) from MDA-MB-231 and MCF-7 cells left untreated, treated with DMSO or 700 nM WA was used for the whole genome amplification (WGA) reaction, enzymatic fragmentation, precipitation and re-suspended in hybridization buffer. Subsequent steps of DNA methylation analysis were carried out according to the standard Infinium HD Assay Methylation Protocol Guide (Part #15019519, Illumina). The BeadChip images were captured using the Illumina iScan. Data quality checks, data normalization and β-value calculation were carried out in the Methylation module of the GenomeStudio software (1.9.0). The raw methylation intensities for each probe were represented as methylation β- values (β = Intensity of the Methylated allele (M)/intensity of the Unmethylated allele (U) + intensity of the Methylated allele (M) + 100). Due to limited number of samples subjected to 450k analyses we filtered out all the probes with detection *p*-value > 0.0001, as well as sex chromosome- and SNP-proximal probes, which have previously been reported to non-specifically bind to other genomic locations [90, 91]. Normalized data sets were used in subsequent analyses for target selection with an arbitrary cut-off of 15% differential methylation. A heatmap of all differentially methylated CpG sites was created using the heatmap.2 function of the ‘gplots’ package in R platform. β-values were represented as row Z-scores by centering and scaling β-values. Target gene heatmaps provided in supplemental information were created by MeV software (TM4, Dana-Farber Cancer Institute, BO, MA, USA). Raw array data were uploaded to the Gene Expression Omnibus (GEO) database and have accession number: GSE97420.

Pathway and netwerk analysis (IPA) was performed in the Ingenuity Pathway Knowledge Base (Ingenuity^®^ Systems, www.ingenuity.com, Qiagen, Hilden, Germany) as described previously [34].

### Pyrosequencing

Forward, biotinylated-reverse and sequencing primers were designed using PyroMark Assay Design 2.0 software. Primer sequences are provided in Supplementary Table 4. Targets of interest were PCR amplified using the PyroMark PCR kit (Qiagen) according to manufacturer's instructions. Successful PCR amplification was evaluated by TBE electrophoresis at 2% agarose gel and visualized by GelRed™ staining. Pyrosequencing was performed using the PyroMark Q24 instrument (Qiagen). Briefly, the biotinylated PCR products were immobilized on streptravidin-coated Sepharose beads (High Performance, GE Healthcare), captured by the PyroMark vacuum Q24 workstation, washed and denatured. Then single stranded PCR products were released into a 24-well plate and annealed to the corresponding sequencing primer for 2 min at 80°C. After the pyrosequencing run was finished, the results were analyzed using the PyroMark Q24 software (Qiagen).

### Epityper mass array

Forward primers with a 10-mer tag sequence and reverse primers that incorporate T7 promoter sequence were designed using the EpiDesigner software available at http://epidesigner.com/. Primer sequences are listed in Supplementary Table 4. PCR was performed using 0.2 U HotStar Taq polymerase (Qiagen), 0.2 μM forward and reverse primers, 200 μM of each dNTP and corresponding PCR buffer (1x). PCR reaction was initiated by 15 min enzyme activation at 95°C and followed by 45 cycles encompassing template denaturation at 94°C for 30 s, annealing at temperatures ranging from 56 to 62°C depending on the primer set for 30 s and elongation at 72°C for 60 s. Final elongation step was performed for 3 min at 72°C. Successful PCR amplification was then evaluated by TBE electrophoresis at 1% agarose gel and visualized by ethidium bromide staining. After SAP treatment, *in vitro* transcription and base-specific cleavage by RNAse A, matrix-assisted laser desorption, ionization-time-of-flight mass spectrometry (Sequenom, San Diego, CA, USA) was applied as described in Ehrich et al. [40].

### Reverse transcription (RT)- PCR and real-time quantitative PCR

1 μg of total RNA, isolated by RNeasy Mini Kit (Qiagen), was reverse transcribed using oligo dT primers (Life Technologies, Praisley, UK) and M-MLV Reverse Transcriptase (Promega, MA, USA). Relative mRNA levels of genes of interest were quantified by real-time quantitative PCR reaction on ABI Prism 7300 (Applied Biosystems) and normalized against the *PPIA* housekeeping gene in case of *ADAM8*, *PLAU*, *TNFSF12*, *ME3* and *GSTM1* genes. *DNMT1*, *DNMT3A*, *DNMT3B* and *JARID1B* mRNA expression levels were normalized against *HPRT* and *ACTB* housekeeping genes as previously described [34]. Sequences of cDNA-specific primers are available upon request. The 2^-ΔΔCt^ method was used for calculation of relative expression levels between samples.

### Western blotting

MDA-MB-231 and MCF-7 cells were treated with WA, or DMSO solvent control as indicated in the figure legends. At the end of the incubation time, total cell lysates were prepared in RIPA buffer. Protein concentrations were determined by the DC protein assay (Bio-Rad, CA, USA). Equal amounts of protein were prepared in SDS-Laemli sample buffer (62.5mM Tris–HCl pH6.8, 2% SDS, 10% glycerol, 0.5% DTT). Proteins were separated on 8.5% SDS-PAGE, and transferred onto a nitrocellulose membrane. Non-specific binding sites on the membrane were blocked with a mixture of 50% Licor blocking buffer (Licor, Lincoln, NA, USA)/50% TBS containing 0.2% Tween-20 for 1 hour. Afterwards membranes were incubated with DNMT1 (MG-261,Imgenex), DNMT3A (#3598, Cell Signaling), DNMT3B (Ab16049, Abcam), JARID1B (HPA027179, Sigma Aldrich), or β-Actin (A5441, Sigma Aldrich) recognizing primary antibodies and visualized with fluorophore-coupled secondary antibody. β-Actin was used as a loading control protein. Detection was performed by use of the Odyssey Imaging System (Licor, Lincoln, NA).

### Chromatin immunoprecipitation (ChIP)

MDA-MB-231 and MCF-7 cells were treated with DMSO or WA as previously described. After 72 h treatment cell culture medium was removed and following two washing steps with PBS protein/DNA complexes were crosslinked by adding formaldehyde directly to the culture medium to a final concentration of 1% for 10 min. After quenching the reaction with 0.65 M glycine, chromatin was extracted from the cell nuclei according to Chromatrap Pro-A protocol (Chromatrap, Wrexham, UK). Protein-DNA complexes were sonicated using the Diagenode Bioruptor (Liége, Belgium) at high settings (12 min, 30 s on/off, 30 min cooling on ice and repeat of the 12 min cycle) and run on 2% agarose gel in 1xTBE to check the shearing efficiency (100–500 bp fragments). 1 μg of sheared chromatin was used for slurry preparation and the same amount was set aside as input. Subsequently, 2:1 ratios of anti-H3K4me3 and mock anti-FLAG control to chromatin were used for immunoprecipitation. Samples were de-crosslinked for two hours at 65°C and proteins were degraded by 1 hour incubation with 0.5 μg Proteinase K at 37°C. After addition of Proteinase K Stop solution DNA samples were used for subsequent qPCR analysis on ABI Prism 7300 (Applied Biosystems). Sequences of the *PLAU* promoter-specific primers are available in Supplementary Table 4. Modified qPCR protocol was used for product amplification. An additional extension step at 80°C for 30 sec was added to the standard qPCR cycling protocol in each of the 40 cycles.

### Statistics

Statistical analysis was performed using GraphPad Prism version 5.00 for Windows (GraphPad Software, La Jolla California, USA) and the computing environment R [92]. Gene-specific pyrosequencing and EpiTyper data before and after WA and DAC treatments were analyzed using a Wilcoxon signed-rank test, showing significance of select pair-wise (control/treatment) comparisons. Values of *p* < 0.05 were considered significant.

Correlations between DNA methylation and different tumor characteristics (HER2+/-, ESR+/-, PR+/- status and TNBC/Luminal B PAM50 intrinsic subtypes) were tested by Wilcoxon signed-rank test in a primary breast tumor dataset retrieved from TCGA [11, 49]. Benjamini-Hochberg adjusted *p* values < 0.05 were considered significant.

For assessing gene expression differences between DMSO, WA or DAC- treated samples (mean ± SEM), as well as the differences in fold enrichment of the H3K4me3 mark at the *PLAU* gene promoter (mean ± SD), one-way ANOVA (Tukey Multiple Comparison test) was used. *p* value < 0.05 was considered significant.

## SUPPLEMENTARY MATERIALS FIGURES AND TABLES




