# Supplementary material for: Epigenetic silencing of triple negative breast cancer hallmarks by Withaferin A
Source: Oncotarget. 2017 Apr 13;8(25):40434–53. doi: 10.18632/oncotarget.17107 (PMC5522326; doi:10.18632/oncotarget.17107)
Supplement: Supplementary file 1 [file oncotarget-08-40434-s001.pdf]

Epigenetic silencing of triple negative breast cancer hallmarks by Withaferin A

Supplementary Materials

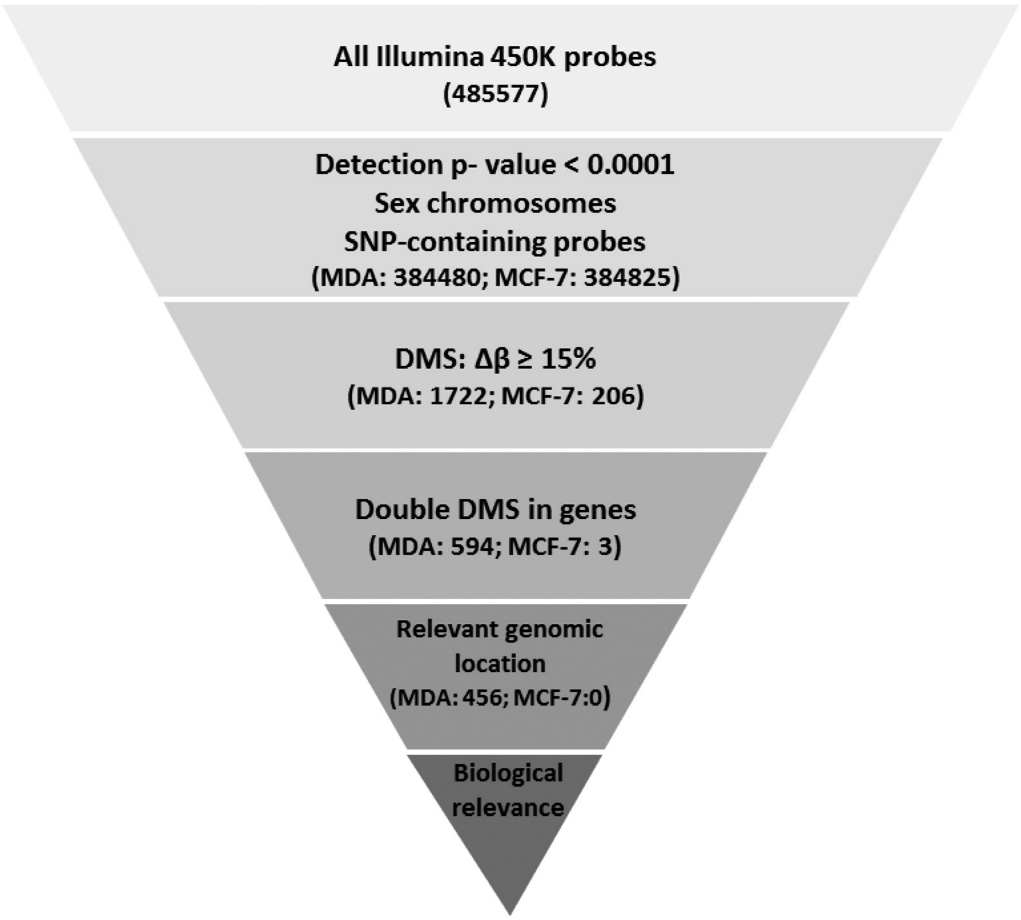

Supplementary Figure 1: Criteria for selection of differentially methylated target genes. β-values of all cg probes were extracted from GenomeStudio (1.9.0) software after data normalization and background subtraction. Cg probes with detection *p*-value > 0.0001 were removed. Additionally, probes mapping to sex chromosomes and SNP-containing probes were removed. Differentially methylated sites (DMS) were defined as those having ≥ 15% difference between solvent control and WA treated samples; Genes of interest had at least two proximal CpG sites with Δβ ≥ 15% in the relevant genomic region (TSS200, TSS1500, CpG island, shore, shelf) and with a known or putative role in the pathogenesis of breast cancer. The number of remaining cg probes for MDA-MB-231 (here named MDA) and MCF-7 cells after each step is indicated.

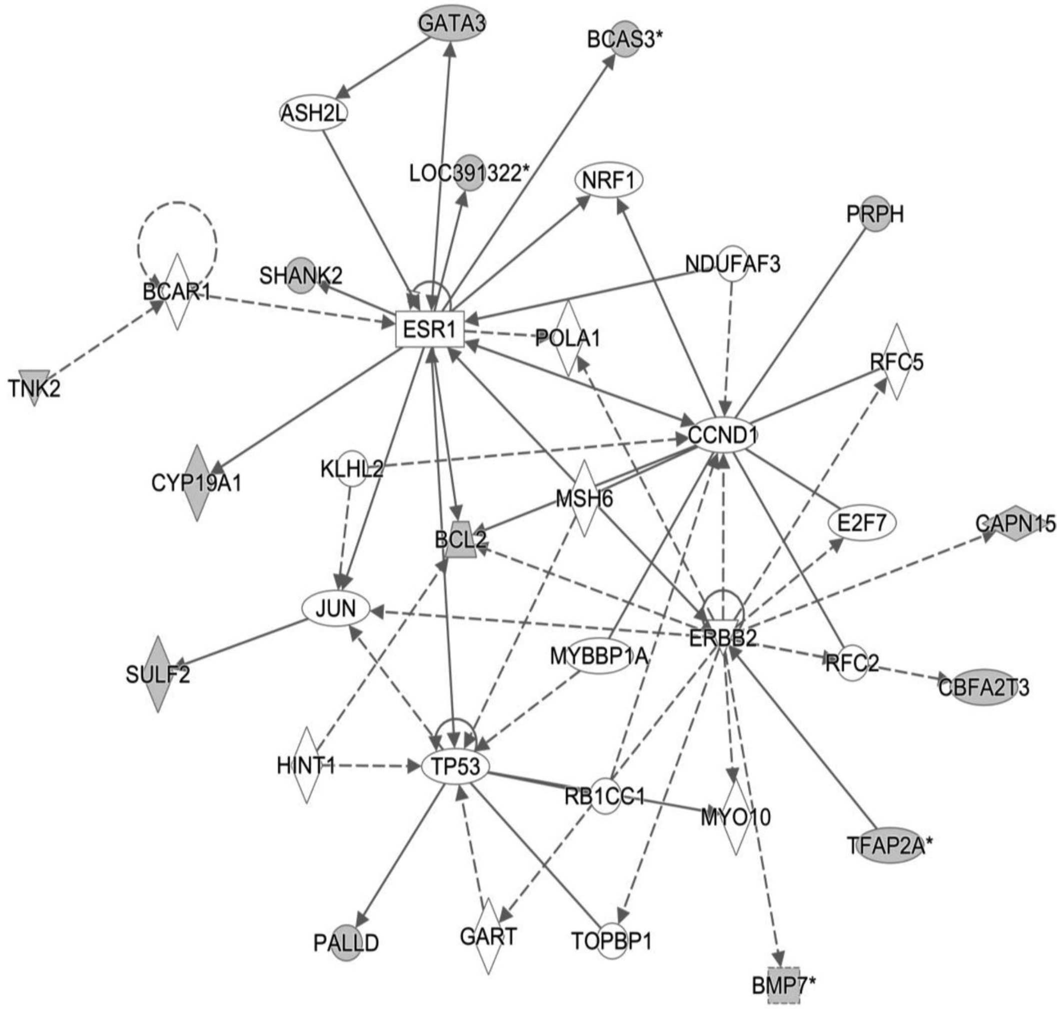

Supplementary Figure 2: IPA network related to cell proliferation and cell motility involving WA-induced CpG methylation changes in MDA-MB-231 cells, which phenocopy methylation pattern in weakly metastatic MCF-7 and normal HMEC cells. Grey nodes represent gene IDs with WA specific methylation alterations in MDA-MB-231 cells, which phenocopy methylation patterns in weakly metastatic MCF-7 and normal HMEC cells . Since differential methylation data were not correlated with gene expression data, no green or red colors are added related to gene silencing or gene activation.

Cell Death and Cell Survival, Network 1

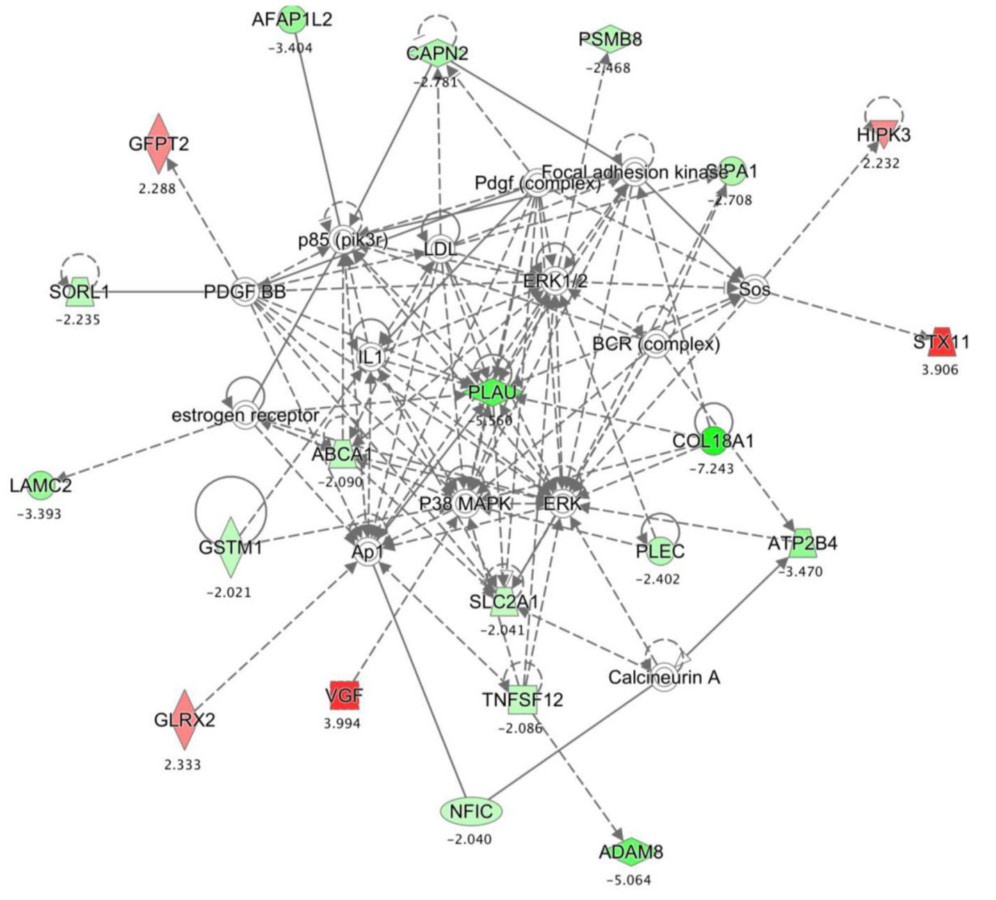

Supplementary Figure 3: IPA network related to Cell Death and Cell Survival based on silenced or activated genes, which are respectively hyper- or hypomethylated, in response to WA in MDA-MB-231 cells. Green nodes are hypermethylated and downregulated; Red nodes are hypomethylated and upregulated. Fold change expression between WA treated and control DMSO sample is indicated below each node.

Cellular Movement, Metastasis, Invasion, Immune Cell Trafficking, Network 2

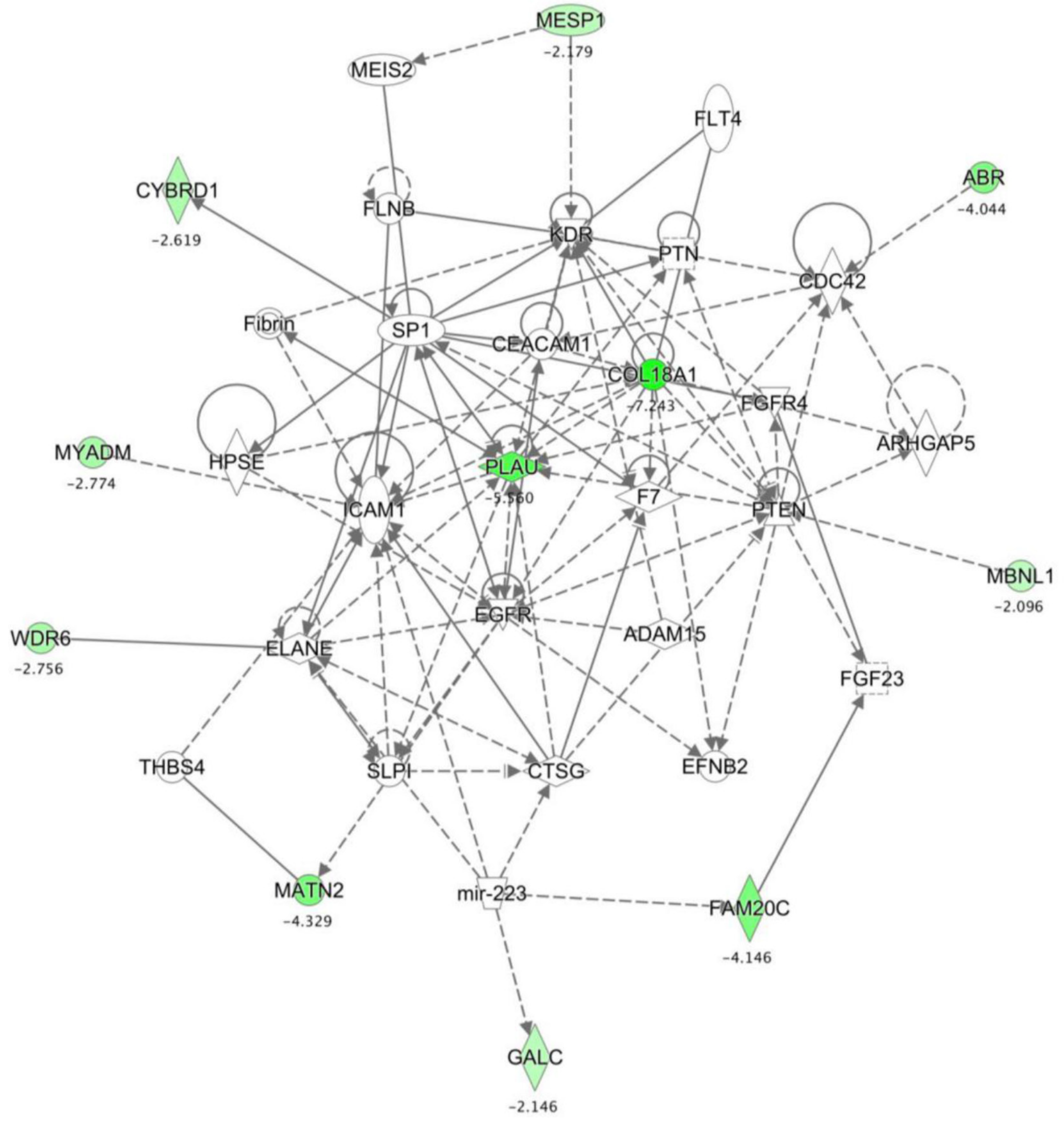

Supplementary Figure 4: IPA network related to Cell Movement, Metastasis, Invasion and Immune Cell Trafficking based on silenced or activated genes, which are respectively hyper- or hypomethylated, in response to WA in MDA-MB-231 cells. Green nodes are hypermethylated and downregulated; Fold change expression between WA treated and control DMSO sample is indicated below each node.

Cellular Growth and Proliferation, Cancer, Network 3

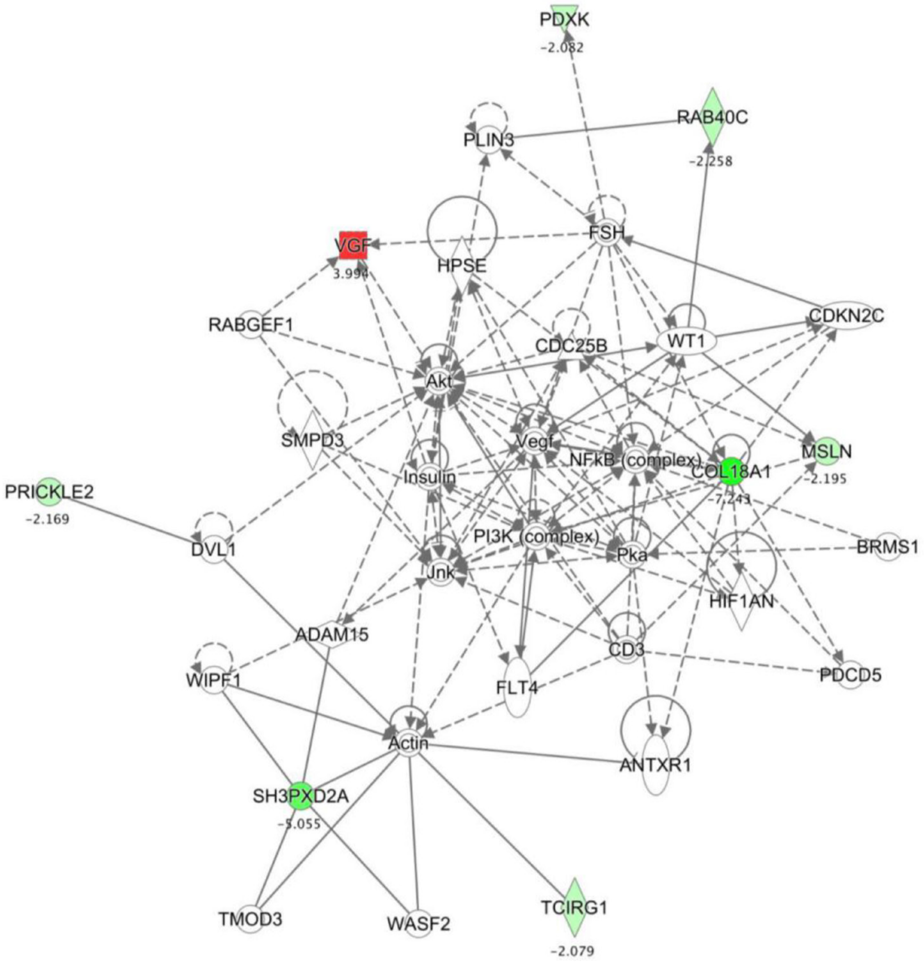

Supplementary Figure 5: IPA network related to Cellular Growth and Proliferation, Cancer based on silenced or activated genes, which are respectively hyper- or hypomethylated, in response to WA in MDA-MB-231 cells. Green nodes are hypermethylated and downregulated; Red nodes are hypomethylated and upregulated. Fold change expression between WA treated and control DMSO sample is indicated below each node.

Cell-To-Cell Signaling and Interaction, Network 4

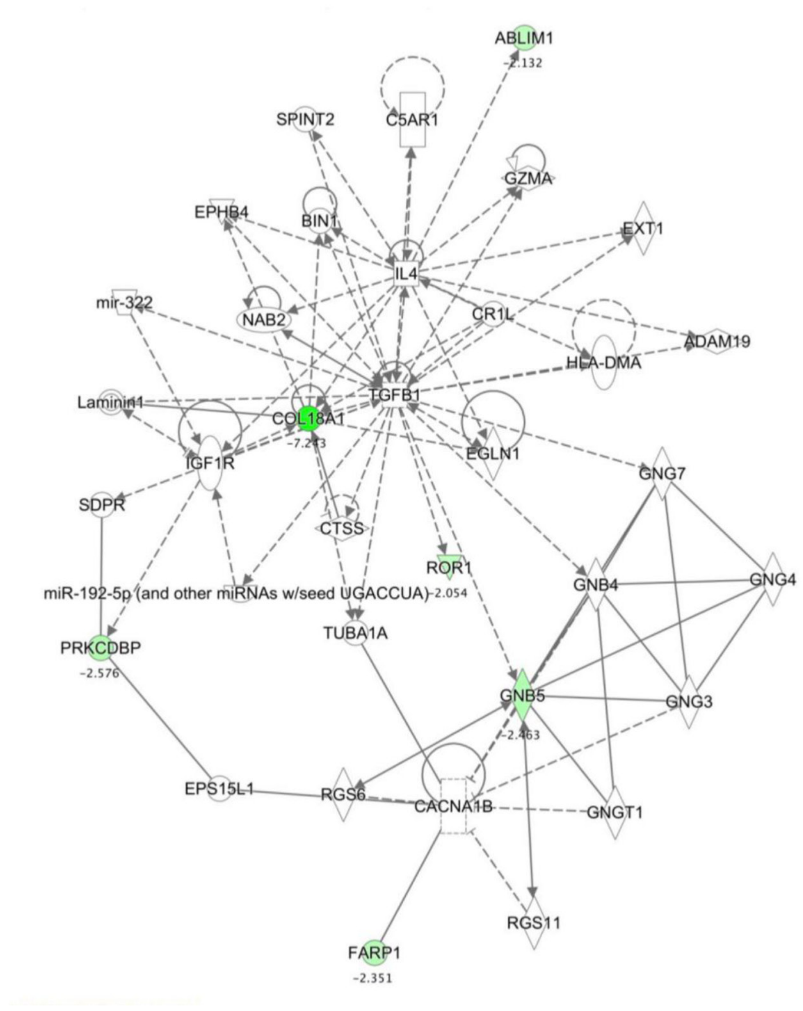

Supplementary Figure 6: IPA network related to Cell-to-Cell Signaling and Interaction based on silenced or activated genes, which are respectively hyper- or hypomethylated, in response to WA in MDA-MB-231 cells. Green nodes are hypermethylated and downregulated; Fold change expression between WA treated and control DMSO sample is indicated below each node.

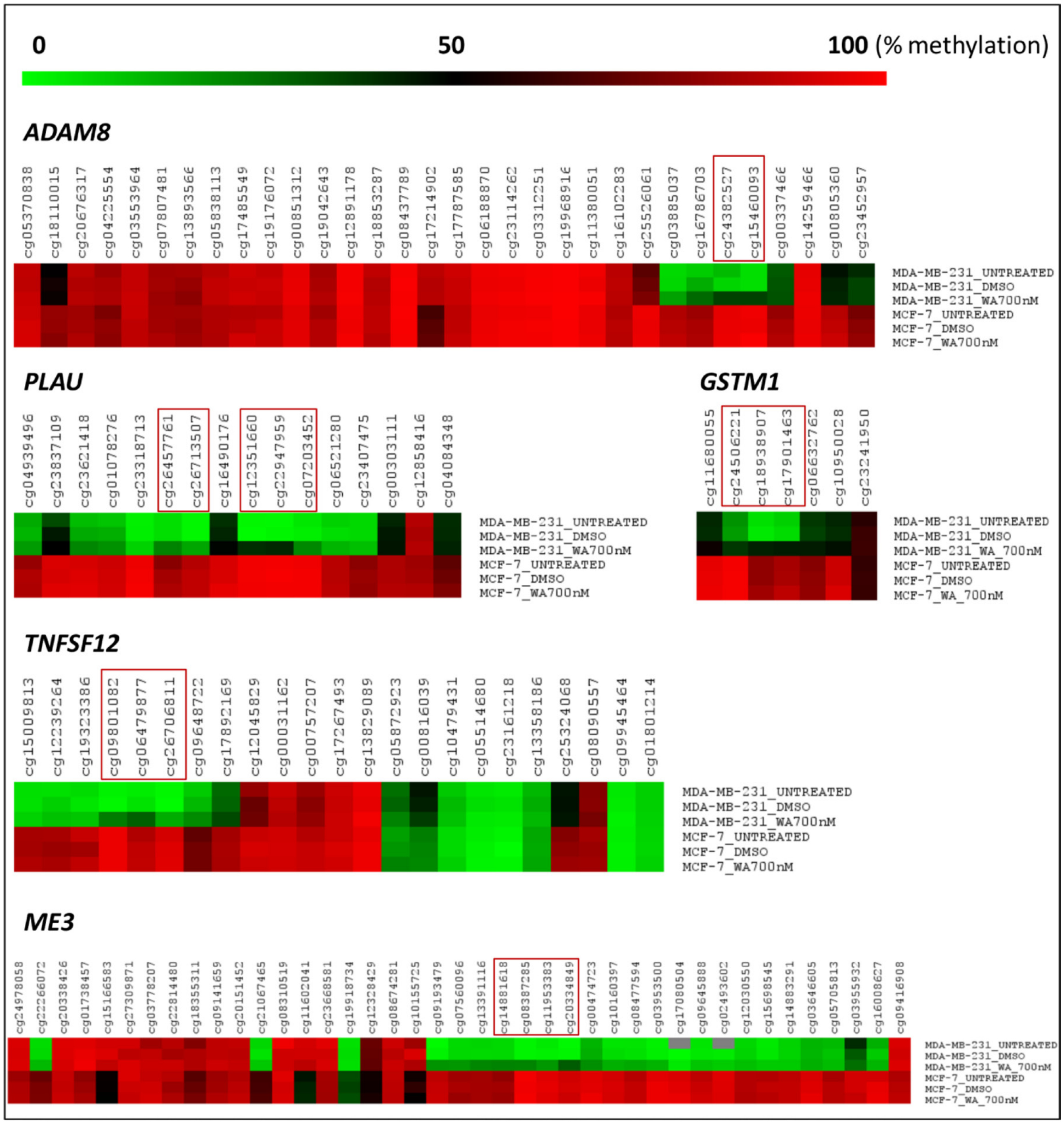

Supplementary Figure 7: The heatmaps illustrate methylation distribution of all 450k cg probes on a 450k BeadChip for selected target genes. Each row represents one sample as indicated on the right side of the heatmap: MDA-MB-231 UNTREATED, DMSO, WA 700 nM, MCF-7 UNTREATED, DMSO, WA 700 nM. Each column represents a unique cg probe within a gene locus. Color gradient on the top from green to red represents level of methylation (from 0 to 100% methylation). Red frames represent the cg probes covered by the pyrosequencing or EpiTyper MassArray assays designed for verification of Illumina 450k BeadChip results.

Supplementary Table 1: Overlap of WA specific silenced or activated genes in MDA-MB-231 cells. which are respectively hyper- or hypomethylated. with cancer hallmarks as determined by IPA-based disease functions

Gene ID name	Δβ MDA WA vs DMSO	FC MDA WA vs DMSO	HALMARKS OF CANCER								
			A	B	C	D	E	F	G	H	I
COL18A1	0.16	-7.24	-		-			-	-		-
PLAU	0.27	-5.56	-	-	-	-		-	-	-	-
ADAM8	0.30	-5.06	-					-	-		
SH3PXD2A	0.21	-5.05	-					-			-
MATN2	0.20	-4.33									
FAM20C	0.16	-4.15									-
ABR	0.20	-4.04									
ATP2B4	0.26	-3.47	-					-			
AFAP1L2	0.19	-3.40			-			-			-
LAMC2	0.16	-3.39	-								
CCDC106	0.22	-2.95									
CAPN2	0.20	-2.78	-		-			-	-	-	-
C2CD2	0.28	-2.78									
MYADM	0.23	-2.77	-								
WDR6	0.45	-2.76									
ITFG1	0.18	-2.72							-		
SIPA1	0.21	-2.71							-		-
CYBRD1	0.17	-2.62									
PRKCDBP	0.16	-2.58			-			-			-
PSMB8	0.25	-2.47						-			-
GNB5	0.19	-2.46									
PLEC1	0.27	-2.40									-
FARP1	0.17	-2.35									-
EPDR1	0.23	-2.35									
RAB40C	0.21	-2.26									
SORL1	0.21	-2.24							-		
WDR36	0.15	-2.23							-		
MSLN	0.19	-2.19			-					-	-
MESP1	0.27	-2.18									-
PRICKLE2	0.24	-2.17			-						-
ME3	0.20	-2.17									
GALC	0.20	-2.15				-					
ABLIM1	0.21	-2.13	-		-						
MBNL1	0.15	-2.10									
ABCA1	0.20	-2.09	-			-		-	-		
TNFSF12	0.22	-2.09	-	-	-	-		-	-	-	-
PDXK	0.29	-2.08							-		
FOXK1	0.15	-2.08			-						-
TCIRG1	0.18	-2.08	-		-				-		-
ROR1	0.17	-2.05	-		-			-	-		-
SLC2A1	0.17	-2.04	-		-	-		-			-
NFIC	0.19	-2.04	-		-			-			-
GSTM1	0.32	-2.02			-	-		-			-
HIPK3	-0.17	2.23									
GFPT2	-0.18	2.29									
GLRX2	-0.24	2.33				+					
STX11	-0.18	3.91									
VGF	-0.22	3.99	+		+			+			+
C5ORF41	-0.15	4.78									

Letters A-I indicate the following cancer hallmarks: A: metastasis. B: angiogenesis. C: cellular proliferation. D: energy metabolism. E: DNA repair. F: cell death. apoptosis. G: inflammatory response. H: cell cycle and DNA replication. I: cellular growth and differentiation. Green color marks decreased gene expression. Red color marks increased gene expression.

Supplementary Table 2: Overlap of WA-specific silenced or activated genes. which are respectively hyper- or hypomethylated. with cancer specific signaling networks related to cell death, cell motility, cell growth and cell-cell signaling

			NETWORKS			
Gene ID name	Δβ MDA WA vs DMSO	FC MDA WA vs DMSO	Cell Death and Cell Survival Network 1	Cellular Movement (invasion. metastasis) Network 2	Cellular Growth and Proliferation Network 3	Cell-To-Cell Signaling and Interaction Network 4
COL18A1	0.16	−7.24	-	-	-	-
PLAU	0.27	−5.56	-	-		
ADAM8	0.30	−5.06	-			
SH3PXD2A	0.21	−5.05				
MATN2	0.20	−4.33		-		
FAM20C	0.16	−4.15		-		
ABR	0.20	−4.04		-		
ATP2B4	0.26	−3.47				
AFAP1L2	0.19	−3.40	-			
LAMC2	0.16	−3.39	-			
CCDC106	0.22	−2.95				
CAPN2	0.20	−2.78	-			
C2CD2	0.28	−2.78				
MYADM	0.23	−2.77		-		
WDR6	0.45	−2.76		-		
ITFG1	0.18	−2.72				
SIPA1	0.21	−2.71	-			
CYBRD1	0.17	−2.62		-		
PRKCDBP	0.16	−2.58				-
PSMB8	0.25	−2.47	-			
GNB5	0.19	−2.46				-
PLEC1	0.27	−2.40				
FARP1	0.17	−2.35				-
EPDR1	0.23	−2.35				
RAB40C	0.21	−2.26				
SORL1	0.21	−2.24	-			
WDR36	0.15	−2.23				
MSLN	0.19	−2.19			-	
MESP1	0.27	−2.18		-		
PRICKLE2	0.24	−2.17				
ME3	0.20	−2.17				
GALC	0.20	−2.15		-		
ABLIM1	0.21	−2.13				-
MBNL1	0.15	−2.10		-		
ABCA1	0.20	−2.09	-			
TNFSF12	0.22	−2.09	-			
PDXK	0.29	−2.08				
FOXK1	0.15	−2.08				
TCIRG1	0.18	−2.08				
ROR1	0.17	−2.05				-
SLC2A1	0.17	−2.04	-			
NFIC	0.19	−2.04	-			
GSTM1	0.32	−2.02	-			
HIPK3	−0.17	2.23	+			
GFPT2	−0.18	2.29	+			
GLRX2	−0.24	2.33	+			
STX11	−0.18	3.91	+			
VGF	−0.22	3.99	+			
C5ORF41	−0.15	4.78				

Green color marks decreased gene expression. Red color marks increased gene expression.

Supplementary Table 3: A list of primary breast tumor samples obtained from TCGA dataset, used for correlation of DNA methylation to intrinsic BC subtypes at select CpG sites in PLAU gene promoter. See Supplementary_Table_3

Supplementary Table 4: Primer sequences

Pyrosequencing primer sets

Gene name	FW Primer	REV Primer	Sequencing primer
ADAM8	GGTTTTTTGGAATT<br>ATTTATTAGGTAGT	[1]TTCCTCACCTATACA<br>AAAAAAAAATACT	GGTAGGGTAGGGGAGTAGA
ME3	TGGGTTAGG<br>GAAGGAGAG	[1]CCCTACCCCCA<br>TCCCTATA	AGGGAAGGAGAGGAT
PLAU	GTAAGATAGG<br>GGAGGGAGT	[1]CTCCCCAACTAT<br>CTCTCTCCT	GGGAGGGTTTGATATAGAGTA
TNFSF12	GGGAGAGGGAGGGGTAAG	[1]CCTTCCTAAATT<br>CCCCACCT	GGAAGTTGTGTGTGATTAAAT
**EpiTyper MassArray primer sets**			
ADAM8	[2]TTTTTTATTTGTG<br>TAAGGGAGGATG	[3]CACCCAAAAACCA<br>CTATACACCTAA	
GSTM1	[2]GTTAGGATTTGG<br>TTGGTGTTTTAAG	[3]ATCCCAATACCCC<br>AATATCATAAAC	
ME3	[2]TTTTGTTAAGAT<br>GGAGAGTTTTAGG	[3]AAACCTAACCAAA<br>AACCAAACAAA	
PLAU	[2]GGGGATAGGT<br>GGATTTTGGT	[3]AATTAAAAAACC<br>CCAAAAAACTTCA	
TNFSF12	[2]GGGGGTTGTGTT<br>TGTTTTAT	[3]TAAAACTCCCTAC<br>CCTTTTCCTAAA	
**Bisulfite-converted DNA control Primers**			
SALL3	GTTTGGGTTTGG<br>TTTTTGTT	ACCCTTTACCAATC<br>TCTTAACTTTC	
**ChIP Primers**			
PLAU	CTCTTGCCCTGA<br>CTTCTCCTTC	CTTGGGCAGCATC<br>AGTCAAAG	
**LEGEND:** [1]-biotin-labeled; [2]-aggaagagag- 10-mer tag; [3]-cagtaatacgactcactatagggagaaggct- T7 promoter sequence			

This table provides the sequences of bisulfite pyrosequencing, EpiTyper MassArray, ChIP, gene editing, and qPCR primers, as well as the bisulfite conversion efficiency control primer set.
