# Supplementary material for: Epigenetic silencing of triple negative breast cancer hallmarks by Withaferin A
Source: Oncotarget. 2017 Apr 13;8(25):40434–53. doi: 10.18632/oncotarget.17107 (PMC5522326; doi:10.18632/oncotarget.17107)
Supplement: Supplementary file 2 [file oncotarget-08-40434-s002.docx]

**Supplementary Table 3. A list of primary breast tumor samples obtained from TCGA dataset, used for correlation of DNA methylation to intrinsic BC subtypes at select CpG sites in *PLAU* gene promoter**

| **Sample_ID** | **Sentrix_ID** | **Sentrix_Position** | **Tissue** | **PAM50** | **Triple.Negative** |
| --- | --- | --- | --- | --- | --- |
| TCGA-A1-A0SM | 6004791006 | R06C02 | Tumor | LumB | LumB |
| TCGA-A2-A04R | 6005486005 | R04C02 | Tumor | LumB | LumB |
| TCGA-A2-A0CT | 6005486023 | R04C02 | Tumor | LumB | LumB |
| TCGA-A2-A0SV | 6004791004 | R01C01 | Tumor | LumB | LumB |
| TCGA-A2-A0SW | 6004791010 | R01C02 | Tumor | LumB | LumB |
| TCGA-A2-A0T4 | 6004791020 | R03C01 | Tumor | LumB | LumB |
| TCGA-A2-A0YG | 6005486005 | R01C02 | Tumor | LumB | LumB |
| TCGA-A2-A0YH | 6005486005 | R06C01 | Tumor | LumB | LumB |
| TCGA-A7-A13F | 6042324030 | R03C02 | Tumor | LumB | LumB |
| TCGA-AN-A0XR | 6005486025 | R06C02 | Tumor | LumB | LumB |
| TCGA-AN-A0XW | 6005486005 | R02C02 | Tumor | LumB | LumB |
| TCGA-AO-A0JC | 6005486014 | R01C01 | Tumor | LumB | LumB |
| TCGA-AO-A0JD | 6005486023 | R03C02 | Tumor | LumB | LumB |
| TCGA-AO-A0JI | 6005486014 | R05C02 | Tumor | LumB | LumB |
| TCGA-AO-A0JM | 6005486019 | R03C01 | Tumor | LumB | LumB |
| TCGA-AO-A12B | 6005486013 | R02C01 | Tumor | LumB | LumB |
| TCGA-AR-A0TQ | 6004791006 | R03C01 | Tumor | LumB | LumB |
| TCGA-AR-A0TT | 6004791010 | R03C02 | Tumor | LumB | LumB |
| TCGA-AR-A0TV | 6004791006 | R06C01 | Tumor | LumB | LumB |
| TCGA-AR-A0TZ | 6004791010 | R03C01 | Tumor | LumB | LumB |
| TCGA-AR-A0U2 | 6005486029 | R06C02 | Tumor | LumB | LumB |
| TCGA-AR-A0U3 | 6005486018 | R05C01 | Tumor | LumB | LumB |
| TCGA-AR-A1AV | 6042324070 | R03C02 | Tumor | LumB | LumB |
| TCGA-B6-A0RL | 6004791004 | R01C02 | Tumor | LumB | LumB |
| TCGA-B6-A0WV | 6005486005 | R05C02 | Tumor | LumB | LumB |
| TCGA-B6-A0WW | 6005486029 | R03C02 | Tumor | LumB | LumB |
| TCGA-B6-A0X5 | 6005486025 | R01C02 | Tumor | LumB | LumB |
| TCGA-BH-A0AU | 6042324030 | R03C01 | Tumor | LumB | LumB |
| TCGA-BH-A0B5 | 6042324037 | R04C01 | Tumor | LumB | LumB |
| TCGA-BH-A0BF | 6042324037 | R02C02 | Tumor | LumB | LumB |
| TCGA-BH-A0BZ | 6042324030 | R01C01 | Tumor | LumB | LumB |
| TCGA-BH-A0C0 | 6005486023 | R06C01 | Tumor | LumB | LumB |
| TCGA-BH-A0C3 | 6042324030 | R06C02 | Tumor | LumB | LumB |
| TCGA-BH-A0DD | 6042324070 | R02C02 | Tumor | LumB | LumB |
| TCGA-BH-A0H0 | 6005486014 | R06C01 | Tumor | LumB | LumB |
| TCGA-BH-A0W3 | 6005486029 | R02C02 | Tumor | LumB | LumB |
| TCGA-C8-A1HG | 6042324048 | R04C01 | Tumor | LumB | LumB |
| TCGA-C8-A1HL | 6042324071 | R01C01 | Tumor | LumB | LumB |
| TCGA-C8-A1HM | 6042324071 | R06C01 | Tumor | LumB | LumB |
| TCGA-E2-A107 | 6005486013 | R01C01 | Tumor | LumB | LumB |
| TCGA-E2-A109 | 6005486017 | R02C02 | Tumor | LumB | LumB |
| TCGA-E2-A10C | 6005486012 | R01C02 | Tumor | LumB | LumB |
| TCGA-E2-A15K | 6042324037 | R06C02 | Tumor | LumB | LumB |
| TCGA-A1-A0SK | 6004791010 | R02C02 | Tumor | Basal | TN |
| TCGA-A1-A0SO | 6004791020 | R02C02 | Tumor | Basal | TN |
| TCGA-A2-A0ST | 6004791006 | R01C02 | Tumor | Basal | TN |
| TCGA-A2-A0SX | 6004791006 | R01C01 | Tumor | Basal | TN |
| TCGA-A2-A0T0 | 6004791020 | R05C02 | Tumor | Basal | TN |
| TCGA-A2-A0T2 | 6004791004 | R04C02 | Tumor | Basal | TN |
| TCGA-A2-A0YM | 6005486005 | R03C01 | Tumor | Basal | TN |
| TCGA-AN-A0XU | 6005486018 | R01C02 | Tumor | Basal | TN |
| TCGA-AO-A0JL | 6005486014 | R02C01 | Tumor | Basal | TN |
| TCGA-AO-A124 | 6005486017 | R03C01 | Tumor | Basal | TN |
| TCGA-AO-A129 | 6005486012 | R04C02 | Tumor | Basal | TN |
| TCGA-AR-A0U4 | 6005486029 | R01C01 | Tumor | Basal | TN |
| TCGA-AR-A1AI | 6042324037 | R03C01 | Tumor | Basal | TN |
| TCGA-AR-A1AQ | 6042324035 | R02C01 | Tumor | Basal | TN |
| TCGA-AR-A1AR | 6042324071 | R02C01 | Tumor | Basal | TN |
| TCGA-AR-A1AY | 6042324037 | R06C01 | Tumor | Basal | TN |
| TCGA-B6-A0IK | 6005486014 | R06C02 | Tumor | Her2 | TN |
| TCGA-B6-A0RE | 6005486023 | R03C01 | Tumor | Basal | TN |
| TCGA-B6-A0RG | 6005486019 | R05C01 | Tumor | LumA | TN |
| TCGA-B6-A0RN | 6004791010 | R05C01 | Tumor | LumA | TN |
| TCGA-B6-A0RS | 6004791004 | R05C01 | Tumor | Her2 | TN |
| TCGA-B6-A0RT | 6004791010 | R04C01 | Tumor | Basal | TN |
| TCGA-B6-A0RU | 6004791010 | R02C01 | Tumor | Basal | TN |
| TCGA-B6-A0WX | 6005486018 | R06C02 | Tumor | Basal | TN |
| TCGA-BH-A0B3 | 6005486021 | R03C01 | Tumor | Basal | TN |
| TCGA-BH-A0B9 | 6005486021 | R06C01 | Tumor | Basal | TN |
| TCGA-BH-A0E0 | 6005486019 | R06C01 | Tumor | Basal | TN |
| TCGA-BH-A0RX | 6004791020 | R06C01 | Tumor | Basal | TN |
| TCGA-BH-A0WA | 6005486005 | R06C02 | Tumor | Basal | TN |
| TCGA-BH-A1EW | 6042324048 | R05C02 | Tumor | LumB | TN |
| TCGA-E2-A14N | 6042324071 | R03C01 | Tumor | Basal | TN |
| TCGA-E2-A1B6 | 6042324072 | R02C01 | Tumor | LumA | TN |
